# Fecal microbiome of horses transitioning between warm-season and cool-season grass pasture within integrated rotational grazing systems

**DOI:** 10.1186/s42523-022-00192-x

**Published:** 2022-06-21

**Authors:** Jennifer R. Weinert-Nelson, Amy S. Biddle, Carey A. Williams

**Affiliations:** 1 Department of Animal Sciences, Rutgers, The State University of New Jersey, New Brunswick, NJ 08901 USA; 2 Department of Animal and Food Sciences, College of Agriculture and Natural Resources, University of Delaware, Newark, DE 19711 USA

**Keywords:** Horse grazing, Equine microbiome, Equine forages, Warm-season grasses

## Abstract

**Background:**

Diet is a key driver of equine hindgut microbial community structure and composition. The aim of this study was to characterize shifts in the fecal microbiota of grazing horses during transitions between forage types within integrated warm- (WSG) and cool-season grass (CSG) rotational grazing systems (IRS). Eight mares were randomly assigned to two IRS containing mixed cool-season grass and one of two warm-season grasses: bermudagrass [*Cynodon dactylon* (L.) Pers.] or crabgrass [*Digitaria sanguinalis* (L.) Scop.]. Fecal samples were collected during transitions from CSG to WSG pasture sections (C–W) and WSG to CSG (W–C) on days 0, 2, 4, and 6 following pasture rotation and compared using 16S rRNA gene sequencing.

**Results:**

Regardless of IRS or transition (C–W vs. W–C), species richness was greater on day 4 and 6 in comparison to day 0 (*P* < 0.05). Evenness, however, did not differ by day. Weighted UniFrac also did not differ by day, and the most influential factor impacting β-diversity was the individual horse (R^2^ ≥ 0.24; *P* = 0.0001). Random forest modeling was unable to accurately predict days within C–W and W–C, but could predict the individual horse based on microbial composition (accuracy: 0.92 ± 0.05). Only three differentially abundant bacterial co-abundance groups (BCG) were identified across days within all C–W and W–C for both IRS (W ≥ 126). The BCG differing by day for all transitions included amplicon sequence variants (ASV) assigned to bacterial groups with known fibrolytic and butyrate-producing functions including members of *Lachnospiraceae*, *Clostridium *sensu stricto* 1, Anaerovorax* the *NK4A214 group* of *Oscillospiraceae*, and *Sarcina maxima*. In comparison, 38 BCG were identified as differentially abundant by horse (W ≥ 704). The ASV in these groups were most commonly assigned to genera associated with degradation of structural carbohydrates included *Rikenellaceae RC9 gut group, Treponema, Christensenellaceae R-7 group*, and the *NK4A214 group* of *Oscillospiraceae*. Fecal pH also did not differ by day.

**Conclusions:**

Overall, these results demonstrated a strong influence of individual horse on the fecal microbial community, particularly on the specific composition of fiber-degraders. The equine fecal microbiota were largely stable across transitions between forages within IRS suggesting that the equine gut microbiota adjusted at the individual level to the subtle dietary changes imposed by these transitions. This adaptive capacity indicates that horses can be managed in IRS without inducing gastrointestinal dysfunction.

**Supplementary Information:**

The online version contains supplementary material available at 10.1186/s42523-022-00192-x.

## Background

Traditional cool-season grass rotational equine pastures in temperate regions of the United States are most productive during the spring, early summer, and fall months, but undergo a period of low forage productivity during hot, dry summer months [1]. Conversely, due to differences in photosynthetic mechanisms, warm-season grasses are most vigorous during this same “summer slump” period, when cool-season grasses are semi-dormant [2–4]. Thus, an integrated rotational grazing system that incorporates both warm- and cool-season grasses can increase pasture yield throughout this period of the growing season [5–7].

Production of prussic acid associated with many warm-season annual species and cold-sensitivity of perennials limit options for integrating warm-season grasses into equine grazing systems. However, some annuals such as teff and improved forage varieties of crabgrass may be grazed by horses with a lesser concern for forage-related disorders (i.e. prussic acid or nitrate toxicity, etc.) [8, 9]. Additionally, cultivation of cold-tolerant bermudagrass varieties has allowed this perennial to be grown in the transition zone corresponding to United States Department of Agriculture (USDA) plant hardiness zones 5–7 [10, 11]. These warm-season annuals and perennials, therefore, could be utilized in equine pastures to increase forage availability during summer months.

While an integrated warm- and cool-season grass rotational grazing management strategy has demonstrated benefits for pasture yield, potential impacts on equine gastrointestinal health have not been evaluated. The equine hindgut microbial ecosystem is particularly sensitive to sudden dietary change [12, 13], as evidenced by the well-documented role of the gut microbiota in carbohydrate induction models of laminitis [14–16]. Additionally, in epidemiological studies, recent dietary change, including changes in hay or forage, has been consistently associated with increased risk of colic [13, 17, 18]. While the role of the hindgut microbiome in the etiology of colic has not been fully elucidated, fluctuations in the gut microbiota preceding and following episodes of colic have been documented [19, 20], and Stewart et al. [21] found differences in the hindgut microbial communities of horses admitted to a veterinary hospital for treatment of colic versus horses admitted for other elective procedures unrelated to gastrointestinal disease. Laminitis is the leading cause of foot lameness in horses [22], and colic is the leading cause of veterinary emergencies and mortality in adult horses [23, 24]. Characterizing shifts in gut microbial community structure that could occur when horses are transitioned between warm- and cool-season grasses and how the equine hindgut microbiota adapts to differing forage types is necessary to fully understand the impact of integrated warm- and cool-season grass rotational grazing management on horse health.

However, a decreased risk of colic has been reported for horses with pasture access [13], potentially suggesting greater stability of the equine hindgut microbiome in grazing horses. Furthermore, the microbial communities of grazing horses have been found to be more diverse than in horses fed mixed diets of conserved forage and concentrate [25]. This research seeks to understand the extent to which this diversity confers enhanced resilience through the transition between forage types for horses managed in integrated rotational grazing systems.

Dietary nutrient composition is a key driver of gut microbial community structure in humans and across animal species including the horse [26–28]. In addition to differences in photosynthetic processes, warm- and cool-season grasses also differ in mechanisms for storage of soluble carbohydrates such as sugars, starches, and fructans [2, 29]. Differences in soluble carbohydrate storage contribute to varying nutrient composition between these two forage types, with non-structural carbohydrate (NSC; NSC = fructans + sugars + starch) concentrations typically greater in cool-season grasses in comparison to warm-season grasses [5, 29, 30]. Prior studies have reported higher fiber and lower protein and digestibility in warm- vs. cool-season grasses [3, 4, 31]; however, the hindgut microbiomes of horses grazing cool- vs. warm-season pasture grasses have not been previously characterized. Recommendations for integrating warm- and cool-season grasses in horse pasture systems should be informed by an understanding of potential impacts of this management practice on the hindgut microbiome. Therefore, the objectives of this study were to characterize shifts in the fecal microbiota of grazing horses during transitions between forage types within integrated warm- and cool-season grass rotational grazing systems.

## Results

### Samples and initial 16s rRNA gene sequence analysis

Fecal samples were collected from eight horses assigned to one of two integrated warm-and cool-season rotational grazing systems containing either the warm-season perennial *Wrangler* bermudagrass [(BER); *Cynodon dactylon* (L.) Pers.; Johnston Seed Company, Enid, OK] or the warm-season annual *Quick-N-Big* crabgrass [(CRB); *Digitaria sanguinalis* (L.) Scop.; Dalrymple Farms, Thomas, OK], with systems denoted as BRS and CRS, respectively. Samples were collected during transitions from cool-season grass to warm-season grass (C–W) and warm-season grass to cool-season grass (W–C). Samples were collected on the day of rotation (D0) and subsequently on D2, D4, and D6 for both C–W and W–C. Prior to each transition, horses had been grazing the respective forage (cool-season grass for C–W and warm-season grass for W–C) for 17–21 days preceding the rotation. The duration of grazing prior to transition was selected to represent the longest duration that horses were likely to continuously consume an individual forage type within the context of integrated warm- and cool-season grass rotational grazing management.

In the 64 fecal samples analyzed for this study, there were a total of 1,289,921 reads prior to initial quality and chimera filtering of paired reads in Qiime 2 [32]. A total of 496,679 reads passed the initial filtering steps. The initial minimum frequency count per sample was 4617 and the maximum was 12,340, with a mean frequency of 7761 and a median frequency of 7750. Following additional filtering to remove low abundance features, 395,520 reads remained, with a minimum frequency of 3711 and a maximum of 10,541. The mean frequency of the final filtered sequence data was 6180, and the median frequency was 6104. The total number of distinct amplicon sequence variants (ASV) generated in this final dataset was 1030. Taxonomy of all ASV included in the final dataset is detailed in Additional File 1.

### Diversity analyses

**Fig. 1:**
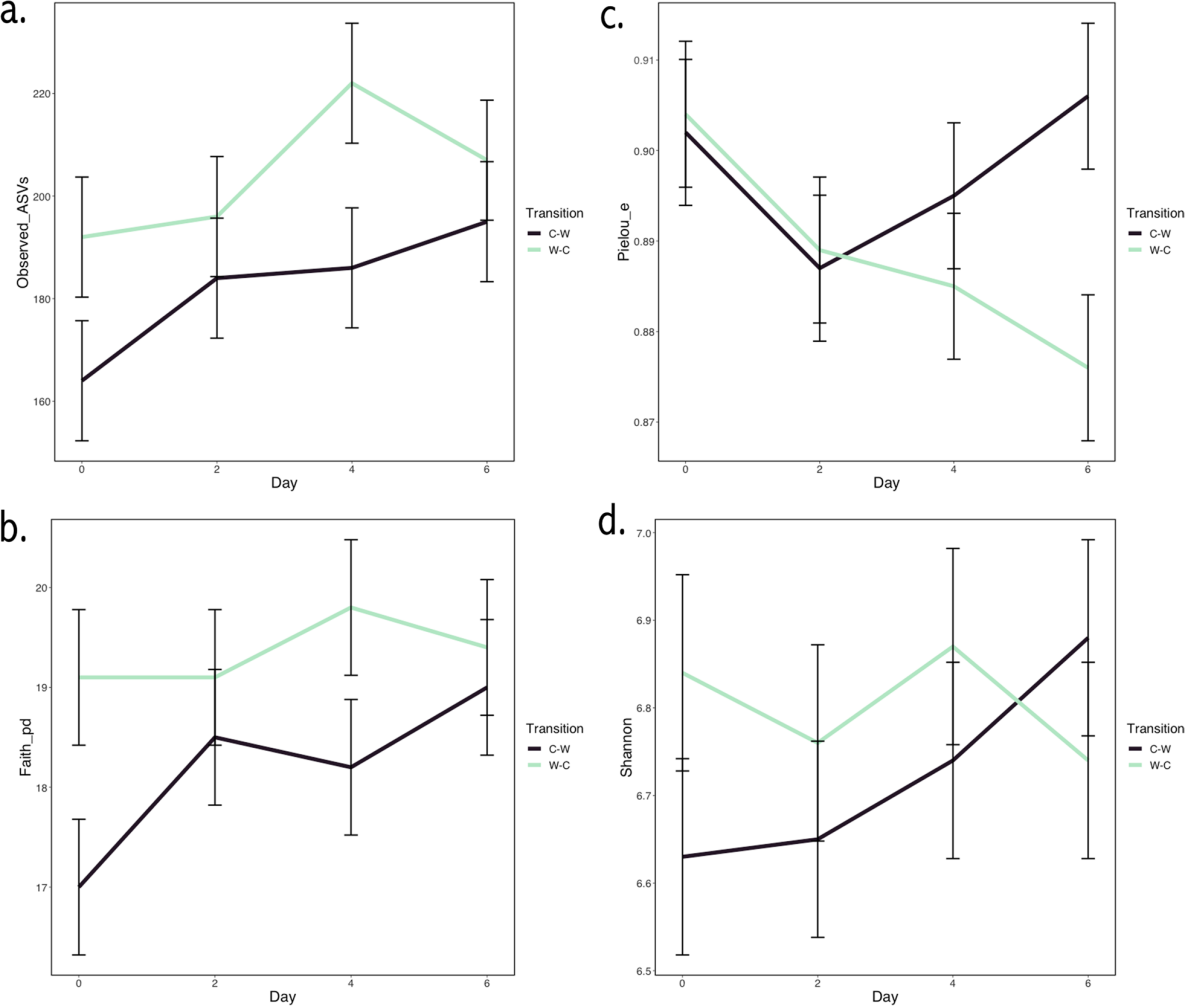
Fecal microbiota α-diversity following transitions between cool-season and warm-season grass. Metrics including **a** Observed ASVs (richness) **b** Pielou’s Evenness, **c** Shannon Diversity Index, and **d** Faith’s Phylogenetic Diversity were analyzed by mixed model ANOVA with Tukey’s post hoc adjustment for transitions from cool-season grass to warm-season grass (C–W) and warm-season grass to cool-season grass (W–C). There was an effect of day and transition for Observed ASVs (*P* < 0.02). There was an effect of transition (*P* = 0.0015), but not for Faith’s Phylogenetic Diversity. Shannon Diversity and Pielou’s Evenness did not differ by transition or day, and there were no significant interactions between any of the main effects for any of the metrics analyzed

For each transition, α-diversity metrics by day, including the Shannon Diversity Index, Faith’s Phylogenetic Diversity, Pielou’s Evenness, and Observed ASVs are shown in Fig. 1a–d. The microbial species richness (Observed ASVs) differed by day and transition (C–W vs. W–C; mixed model AOVA with Tukey’s post hoc adjustment; *P* < 0.02) but did not differ by grazing system. Regardless of system or transition, species richness was greater on D4 and D6 in comparison to D0 (*P* < 0.05). Overall, species richness was greater in W–C than in C–W transitions (*P* = 0.0008). Evenness, however, did not differ by day or transition, but there was a trend for difference by system, with evenness greater in CRS vs. BRS (*P* < 0.10). The Shannon Diversity Index did not vary by day, transition, or grazing system. Faith’s Phylogenetic Diversity differed by transition, with greater diversity in W–C vs. C–W (*P* = 0.0015). There was also a trend for a difference in Faith’s Diversity by day, with greater diversity on D6 in comparison to D0 (*P* = 0.07). There were no interactions between main effects terms for any of the α-diversity metrics evaluated.

**Fig. 2:**
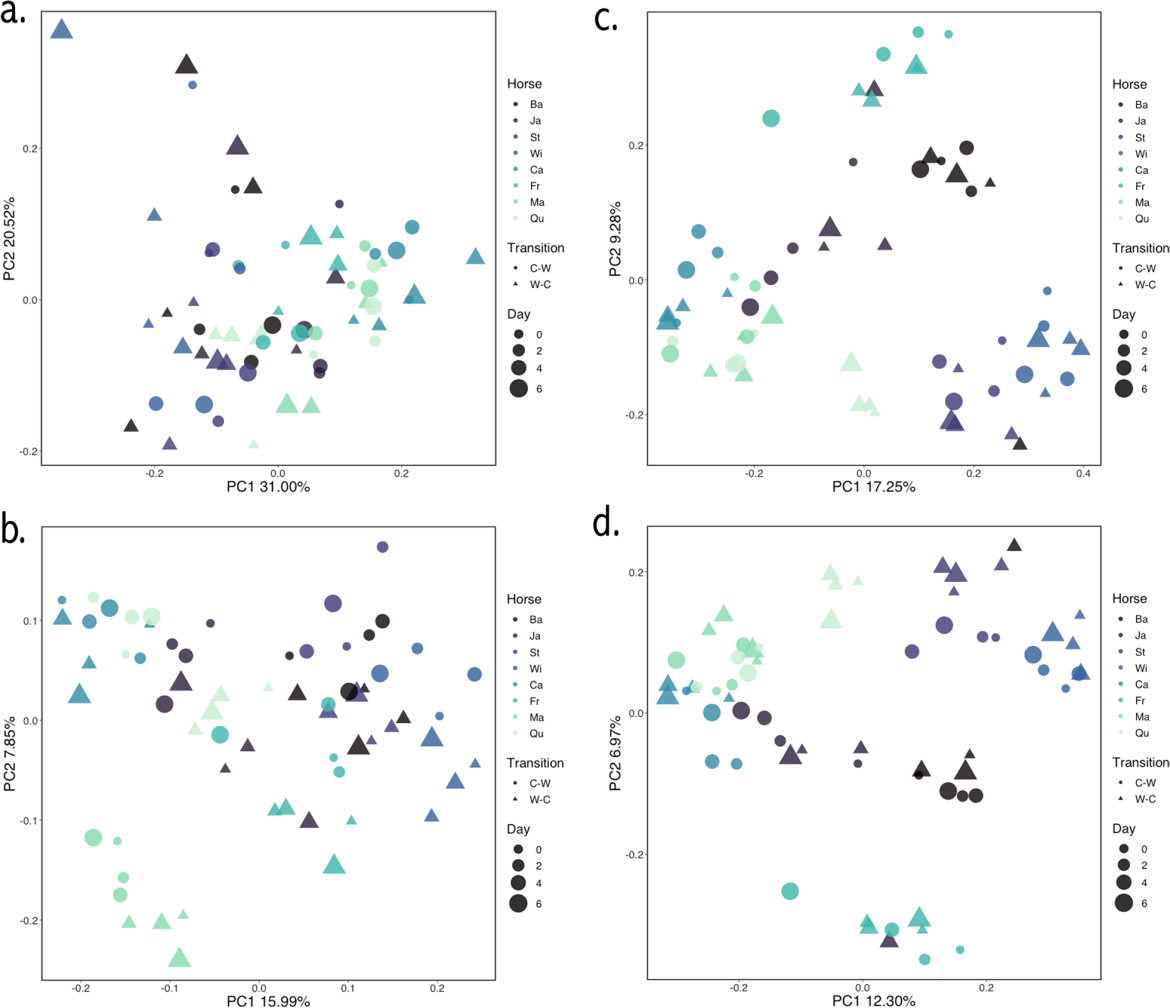
Fecal microbiota β-diversity following transitions between cool-season and warm-season grass. Metrics including **a** Weighted UniFrac, **b** Unweighted UniFrac, **c** Bray Curtis Dissimilarity, and **d** Jaccard Index were analyzed for transitions from cool-season to warm-season grass (C–W) and warm-season to cool-season grass (W–C) in both the bermudagrass integrated rotational grazing system (BRS) and the crabgrass integrated system (CRS). Individual horses are designated with two-letter abbreviations and arranged such that horses on the darker end of the color spectrum grazed in BRS (n = 4) and horses on the lighter end of the color spectrum grazed in CRS (n = 4). Analysis by PERMANOVA with the Adonis action in Qiime 2 (v.2020.8) found that the influence of individual horse was the most influential factor shaping β-diversity across all metrics (R^2^ ≥ 24; *P* = 0.001)

Principle coordinate analysis of β-diversity metrics did not reveal distinct clustering by day within each transition (Fig. 2a–d). This was confirmed with statistical analysis by Adonis PERMANOVA, which did not find a significant effect of day for Weighted UniFrac (R^2^ = 0.013; *P* = 0.13). There was, however, as significant effect of day for Unweighted UniFrac (R^2^ = 0.013; *P* = 0.039), as well as for both Bray–Curtis Dissimilarity (R^2^ = 0.014; *P* = 0.014) and Jaccard Index (R^2^ = 0.015; *P* = 0.011). There was also a significant effect of transition (C–W vs. W–C) for Weighted UniFrac (R^2^ = 0.032), Unweighted UniFrac (R^2^ = 0.052), Bray–Curtis (R^2^ = 0.037), and Jaccard (R^2^ = 0.042; *P* = 0.001). The influence of grazing system (Weighted UniFrac: R^2^ = 0.16; Unweighted UniFrac: R^2^ = 0.10; Bray–Curtis: R^2^ = 0.11; Jaccard: R^2^ = 0.081; *P* = 0.001) was stronger than the influence of either day or transition. However, the most influential factor was the individual horse, with strong R^2^ values for all β-diversity metrics (Weighted UniFrac: R^2^ = 0.24; Unweighted UniFrac: R^2^ = 0.31; Bray–Curtis: R^2^ = 0.34; Jaccard: R^2^ = 0.29; *P* = 0.001), which was also reflected in visual clustering in the PCoA plots. There were also significant interactions between main terms, which are detailed in full in Table 1. Subsequent application of PERMDISP (permutational analysis of dispersion) confirmed that differences in the main effects of day, transition, grazing system, and horse were not due to differences of variance or dispersion within groups.

**Table 1:** Results of Adonis^1^ multivariate PERMANOVA analysis of β-diversity showing influence of main effects and interactions

Model effects/interactions	β-diversity metric							
	Weighted UniFrac		Unweighted UniFrac		Bray–Curtis Dissimilarity		Jaccard index	
	R^2^	*P*-value	R^2^	*P*-value	R^2^	*P*-value	R^2^	*P*-value
Day^2^	0.013	0.133	0.013	0.039	0.014	0.014	0.015	0.011
Transition^3^	0.032	0.001	0.052	0.001	0.037	0.001	0.042	0.001
Grazing system^4^	0.161	0.001	0.097	0.001	0.110	0.001	0.081	0.001
Horse	0.242	0.001	0.310	0.001	0.336	0.001	0.286	0.001
Day*transition	0.039	0.001	0.017	0.003	0.023	0.001	0.019	0.002
Day*grazing system	0.011	0.159	0.010	0.092	0.012	0.032	0.012	0.044
Day*horse	0.062	0.123	0.051	0.161	0.050	0.098	0.061	0.015
Transition*grazing system	0.024	0.011	0.023	0.003	0.024	0.001	0.025	0.001
Transition*horse	0.073	0.021	0.111	0.001	0.086	0.001	0.109	0.001
Day*transition*grazing system	0.035	0.002	0.012	0.040	0.018	0.003	0.015	0.004
Day*transition*horse	0.062	0.109	0.581	0.035	0.059	0.007	0.066	0.004

^1^Analyses were conducted in Qiime2 (v.2020.8) (Boylen et al., 2019)

^2^Samples were collected on day 0, 2, 4, 6 of each transition

^3^Samples were collected for transitions from cool-season to warm-season pasture grass and from warm- to cool-season grass

^4^Horses were managed in two integrated systems, one of which contained *Quick-N-Big* crabgrass as the warm-season forage and the other contained *Wrangler* bermudagrass

### Differential abundance

Application of Sparce Cooccurrence Network Investigation for Compositional Data (SCNIC) identified 149 distinct bacterial co-abundance groups (BCG), with 621 individual ASV remaining ungrouped. Random forest classification models were able to accurately predict grazing system (0.95 ± 0.06), transition (0.97 ± 0.06), and horse (0.92 ± 0.05) based on microbial composition. However, day could not be accurately predicted in either the C–W (0.25 ± 0.13) or the W–C transitions (0.22 ± 0.18). Accordingly, Analysis of Composition of Microbes (ANCOM) found three BCG/ungrouped ASV that differed by transition (W ≥ 264), seven BCG that differed by grazing system (W ≥ 701), and 42 BCG and ungrouped ASV that differed across horses (W ≥ 704). Conversely, there was only one differentially abundant BCG across days within each of the transitions (C–W and W–C) for horses in CRS (W ≥ 126). There was also only one BCG that differed across days within the W–C transition for horses in BRS (≥ 299), while no BCG or ungrouped ASV differed across days within the C–W transition for BRS based on analysis by ANCOM.

The BCG and ungrouped ASV identified as differing by grazing system, transition, or day as well as taxonomic classifications are presented in Tables 2, 3 and 4. Features differing across horses, including taxonomy, are detailed in Additional File 2. The ungrouped ASV and ASV within BCG that differed by transition were assigned to taxa including the genera *Christensenellaceae R-7 group* and *Rikenellaceae RC9 gut group* as well as ASV assigned only to the family level for *Lachnospiraceae*, and the class level for *Bacilli* and *Clostridia*. These ungrouped ASV and BCG were all more abundant in the C–W transition, but their summed relative abundance comprised < 1% of total microbial community on average across all samples. The BCG that differed between grazing systems also represented a small portion of the total microbial community at < 7%. Of these BCG, five were more abundant in BRS. The BCG that were more abundant in BRS included ASV members assigned to the genera *Fibrobacter, Treponema, Christensellaceae R-7 group, Prevotellaceae 6a6A1 group, Methanocorpusculum,* the *NK4A214 group* within *Oscillospiraceae*, and the *UCG-010* genus and family within *Oscillospirales*. Additional ASV within these groups were mapped only to the order level for *Bacteroidales* and the class level for *Bacteroidia*. Two BCG were more abundant in CRS, with ASV members from *Denitrobacterium detoxificans* and the genera *Desulfovibrio* and *Phascolarctobacterium*.

**Table 2:** Differentially abundant^1^ bacterial co-abundance groups (BCG)^2^ and ungrouped amplicon sequence variants (ASV) across transitions^3^

BCG	W ^4^	Taxonomic lineage ^5^
BCG_76	264	*p__Firmicutes; c__Clostridia; o__Lachnospirales; f__Lachnospiraceae* *p__Firmicutes; c__Clostridia*
BCG_85	272	*p__Firmicutes; c__Clostridia; o__Christensenellales; f__Christensenellaceae; g__Christensenellaceae _R-7_group* *p__Bacteroidota; c__Bacteroidia; o__Bacteroidales; f__Rikenellaceae; g__Rikenellaceae_RC9_gut_group; s__uncultured_Rikenella*
ASV_85	277	*p__Firmicutes; c__Bacilli*

^1^Differential abundance was analyzed by Analysis of Composition of Microbes (ANCOM) in Qiime 2 (v.2020.8)

^2^The ASV were grouped into BCG using Sparce Cooccurrence Network Investigation for Compositional Data in Qiime 2 (v.2020.8)

^3^Transitions from cool-season to warm-season grass versus from warm-season to cool-season grass

^4^For ANCOM, H_0(*ij*)_: mean(log[x_*i*_/x_*j*_) = mean(log[y_*i*_/y_*j*_). The W value indicates the number of times H_0(*ij*)_ is rejected for the *ith* species

^5^Taxonomic assignment was conducted using the most recent SILVA database (SSU 138)

**Table 3:** Differentially abundant^1^ bacterial co-abundance groups (BCG)^2^ and ungrouped amplicon sequence variants (ASV) across grazing systems^3^

BCG	W ^4^	Taxonomic lineage ^5^
BCG_7	762	*p__Firmicutes; c__Clostridia; o__Christensenellales; f__Christensenellaceae; g__Christensenellaceae _R-7_group; s__uncultured_Christensenella* *p__Spirochaetota; c__Spirochaetia; o__Spirochaetales; f__Spirochaetaceae; g__Treponema* *p__Bacteroidota; c__Bacteroidia; o__Bacteroidales; f__Prevotellaceae; g__Prevotellaceae_Ga6A1_group; s__unidentified_rumen* *p__Firmicutes; c__Clostridia; o__Christensenellales; f__Christensenellaceae; g__Christensenellaceae _R-7_group*
BCG_21	752	*p__Firmicutes; c__Clostridia; o__Oscillospirales; f__[Eubacterium]_coprostanoligenes_group; g__[Eubacterium]_coprostanoligenes_group* *p__Firmicutes; c__Clostridia; o__Oscillospirales; f__Oscillospiraceae; g__NK4A214_group; s__unidentified_rumen* *p__Firmicutes; c__Clostridia; o__Oscillospirales; f__UCG-010; g__UCG-010; s__uncultured_eubacterium*
BCG_43	752	*p__Bacteroidota; c__Bacteroidia; o__Bacteroidales; f__Prevotellaceae; g__Prevotellaceae_Ga6A1_group; s__unidentified_rumen* *p__Fibrobacterota; c__Fibrobacteria; o__Fibrobacterales; f__Fibrobacteraceae; g__Fibrobacter; s__bacterium_MB2022* *p__Bacteroidota; c__Bacteroidia; o__Bacteroidales; f__Prevotellaceae; g__Prevotellaceae_Ga6A1_group; s__unidentified_rumen*
BCG_82	756	*p__Desulfobacterota; c__Desulfovibrionia; o__Desulfovibrionales; f__Desulfovibrionaceae; g__Desulfovibrio* *p__Actinobacteriota; c__Coriobacteriia; o__Coriobacteriales; f__Eggerthellaceae; g__Denitrobacterium; s__Denitrobacterium_detoxificans*
BCG_91	701	*p__Firmicutes; c__Clostridia; o__Oscillospirales; f__Oscillospiraceae; g__NK4A214_group* *p__Halobacterota; c__Methanomicrobia; o__Methanomicrobiales; f__Methanocorpusculaceae; g__Methanocorpusculum*
BCG_104	701	*p__Bacteroidota; c__Bacteroidia; o__Bacteroidales* *p__Bacteroidota; c__Bacteroidia*
BCG_141	712	*p__Firmicutes; c__Negativicutes; o__Acidaminococcales; f__Acidaminococcaceae; g__Phascolarctobacterium; s__wallaby_gut* *p__Firmicutes; c__Negativicutes; o__Acidaminococcales; f__Acidaminococcaceae; g__Phascolarctobacterium*

^1^Differential abundance was analyzed by Analysis of Composition of Microbes (ANCOM) in Qiime 2 (v.2020.8)

^2^The ASV were grouped into BCG using Sparce Cooccurrence Network Investigation for Compositional Data in Qiime 2 (v.2020.8)

^3^The bermudagrass integrated rotational grazing system versus the crabgrass integrated rotational grazing system

^4^For ANCOM, H_0(*ij*)_: mean(log[x_*i*_/x_*j*_) = mean(log[y_*i*_/y_*j*_). The W value indicates the number of times H_0(*ij*)_ is rejected for the *ith* species

^5^Taxonomic assignment was conducted using the most recent SILVA database (SSU 138)

**Table 4:** Differentially abundant^1^ bacterial co-abundance groups (BCG)^2^ and ungrouped amplicon sequence variants (ASV) across days^3^

BCG	W ^4^	Taxonomic lineage ^5^
BCG_114 ^6^	126	*p__Firmicutes; c__Clostridia; o__Lachnospirales; f__Lachnospiraceae* *p__Firmicutes; c__Clostridia; o__Oscillospirales; f__Oscillospiraceae; g__NK4A214_group*
BCG_124 ^7^	253	*p__Firmicutes; c__Clostridia; o__Clostridiales; f__Clostridiaceae; g__Sarcina; s__Sarcina_maxima* *p__Firmicutes; c__Clostridia; o__Clostridiales; f__Clostridiaceae; g__Sarcina; s__Sarcina_maxima*
BCG_10 ^8^	299	*p__Firmicutes; c__Clostridia; o__Clostridiales; f__Clostridiaceae; g__Clostridium_sensu_stricto_1* *p__Firmicutes; c__Clostridia; o__Clostridiales; f__Clostridiaceae; g__Clostridium_sensu_stricto_1* *p__Firmicutes; c__Clostridia; o__Lachnospirales; f__Lachnospiraceae* *p__Firmicutes; c__Clostridia; o__Peptostreptococcales-Tissierellales; f__Anaerovoracaceae; g__Anaerovorax*

^1^Differential abundance was analyzed by Analysis of Composition of Microbes (ANCOM) in Qiime 2 (v.2020.8)

^2^The ASV were grouped into BCG using Sparce Cooccurrence Network Investigation for Compositional Data in Qiime 2 (v.2020.8)

^3^Samples were collected on days 0, 2, 4, and 6 of each transition (cool-season grass to warm-season grass [C–W] and warm-season grass to cool-season grass [W–C])

^4^For ANCOM, H_0(*ij*)_: mean(log[x_*i*_/x_*j*_) = mean(log[y_*i*_/y_*j*_). The W value indicates the number of times H_0(*ij*)_ is rejected for the *ith* species

^5^Taxonomic assignment was conducted using the most recent SILVA database (SSU 138)

^6^Differed across days in the C–W transition within the crabgrass integrated rotational grazing system (CRS)

^7^Differed across days in the W–C transition within CRS

^8^Differed across days in the W–C transition within the bermudagrass integrated rotational grazing system (BRS). There were no differentially abundant features across days in the C–W transition for BRS

**Fig. 3:**
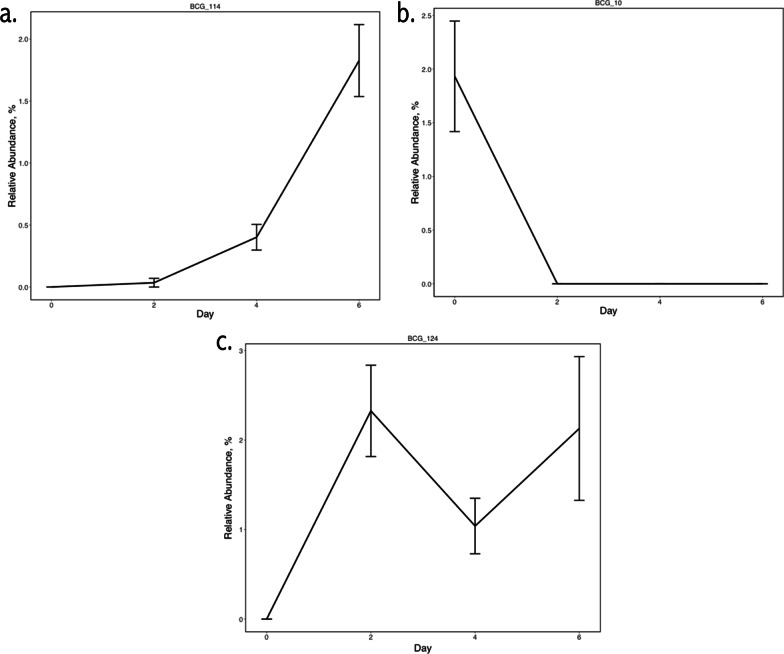
Differentially abundant bacterial co-abundance groups (BCG) following transitions between cool-season and warm-season grass. Relative abundances are shown across days within transitions between **a** cool-season grass to warm-season grass pasture and **b** warm-season to cool-season grass within the crabgrass integrated rotational grazing system and the **c** warm-season to cool-season grass transition within the bermudagrass integrated rotational grazing system. The BCG were identified as differentially abundant using Analysis of Composition of Microbes (ANCOM) in Qiime 2 (v.2020.8) (W ≥ 126)

Similarly, the three BCG that differed by day within transitions and systems comprised a small percentage (< 2%) of the total microbial community. The BCG that differed by day for the C–W transition within CRS (BCG_114) included ASV mapped to the *Lachnospiraceae* family and the *NK4A214 group* within *Oscillospiraceae,* with this BCG increasing across days within C–W (Fig. 3a). The BCG that differed by day (increasing) for the W–C transition within CRS (BCG_124; Fig. 3b) contained two ASV assigned to *Sarcina maxima*, while the BCG that differed by day (decreasing) for the W–C transition within BRS (BCG_10; Fig. 3c) included member ASV assigned to *Clostridium *sensu stricto* 1, Anaerovorax,* and *Lachnospiraceae.*

**Fig. 4:**
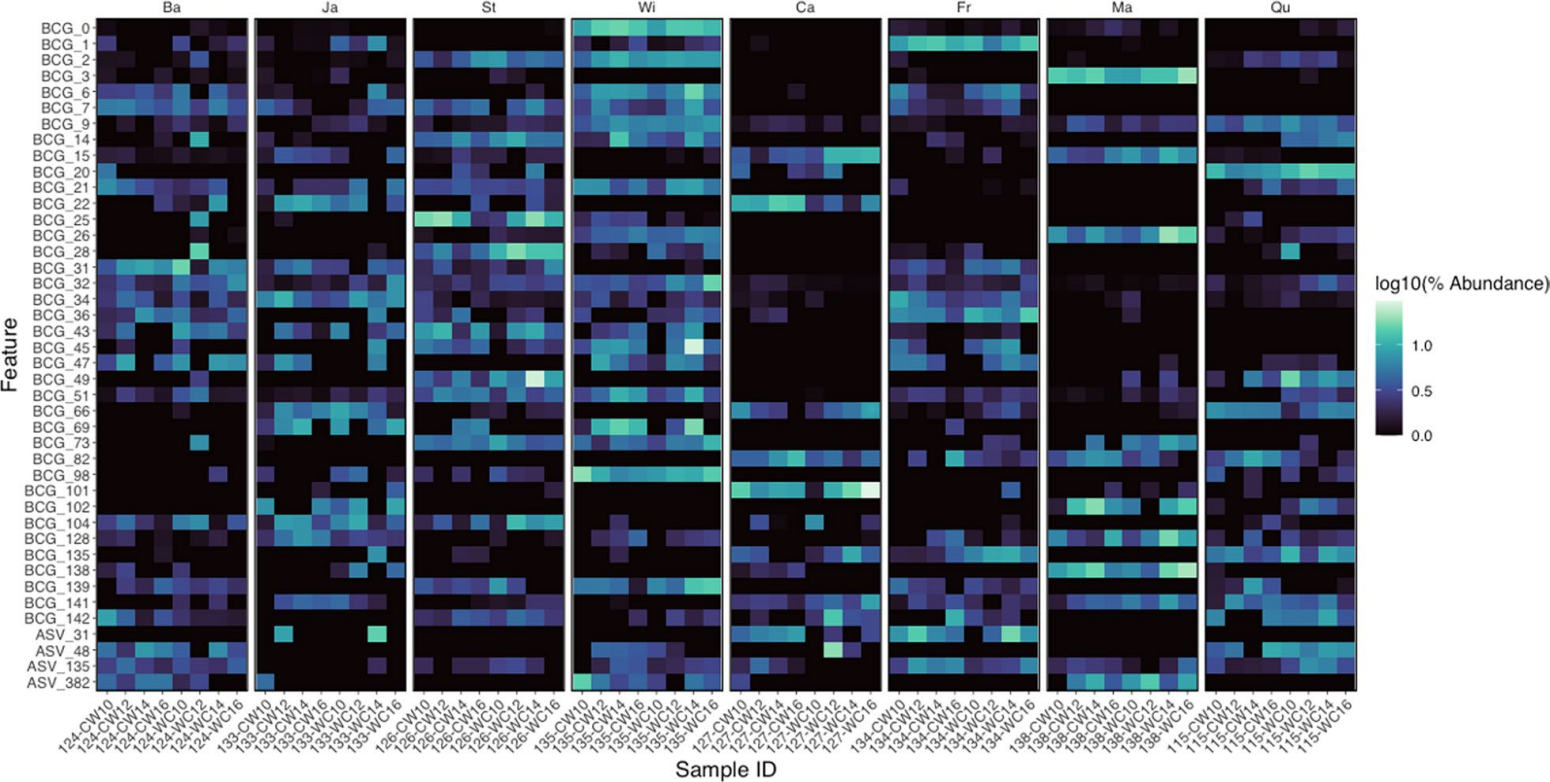
Differentially abundant bacterial co-abundance groups (BCG) and ungrouped amplicon sequenc variants (ASV) by horse. Features (BCG and ASV) were dentified as differentially abundant (W ≥ 704) across horses using Analysis of Composition of Microbes (ANCOM) in Qiime 2 (v.2020.8). Individual horses are designated with two-letter abbreviations and arranged such that each horse is represented by an individual facet grid, with horses on the left-hand side assigned to the bermudagrass integrated rotational grazing system (n = 4) and horses on the right-hand side assigned to the crabgrass integrated system (n = 4). Sample IDs on the x-axis are arranged such that the first four samples within each horse represent transitions from cool-season grass to warm-season grass and the final four samples within each horse represent transitions from warm-season grass to cool-season grass. Within transitions, samples are arranged from D0 through D6

In comparison to the relatively small number of BCG for which abundance changed in response to transition, system, and day, 38 BCG and 4 ungrouped ASV were identified as differentially abundant by horse. When averaged across all samples, the BCG and ungrouped ASV that differed by horse comprised > 32% of the total microbial community. Abundance of BCG and ungrouped ASV identified as differing by horse are shown in Fig. 4. The genera to which these ungrouped ASV and ASV members of the BCG were most commonly assigned included *Rikenellaceae RC9 gut group* (18 ASV), *Treponema* (10 ASV), *Christensenellaceae R-7 group* (10 ASV), and the *NK4A214 group* of *Oscillospiraceae* (8 ASV). Shifts in relative abundance of the twelve most abundant BCG that differed by horse are shown in Additional File 3.

Analysis of microbial composition at the genus-level confirmed results presented for BCG analysis. Grazing system, transition, and horse could be predicted based on microbial composition through random forest classification with accuracies ≥ 0.80, while day could not be accurately predicted for either transition (accuracy ≤ 0.22). Additionally, ANCOM identified a limited number of genera groups and/or ungrouped genera as differentially abundant by grazing system, transition, and day (within transition for each system) in comparison to genera groups that differed by horse (see Additional File 4). However, predictive accuracy of the random forest classifiers was over 10% lower than for the BCG analysis and the percentage of the total microbial communities represented by the differential abundant genera was also lower (see analytical comparison in Additional File 5).

### Fecal pH

**Fig. 5:**
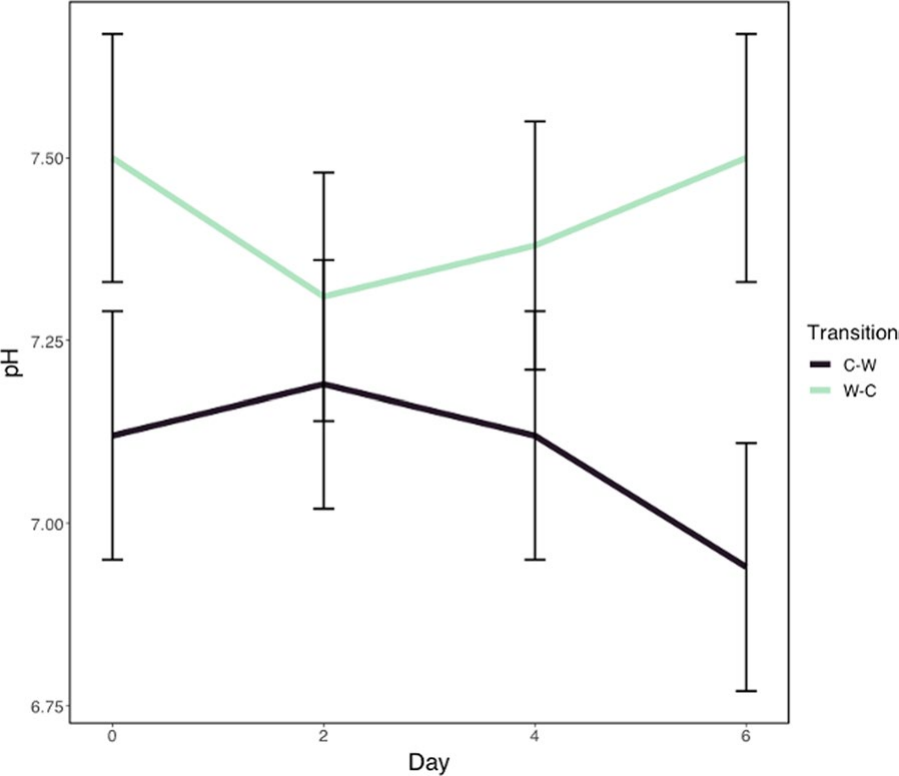
Fecal pH following transitions between cool-season and warm-season grass. Fecal pH of horses across six days following transitions from cool-season to warm-season grass (C–W) and warm-season to cool-season grass (W–C) in both the bermudagrass integrated rotational grazing system and the crabgrass integrated system. There was an effect of transition and grazing system (mixed model ANOVA with Tukey’s post hoc adjustment; *P* ≤ 0.02), but pH did not differ by day. There were no significant interactions between main effects

Fecal pH across the days of each transition is shown in Fig. 5. Consistent with above results for microbiome data, fecal pH differed by transition and grazing system (mixed model AOVA with Tukey’s post hoc adjustment; *P* ≤ 0.02), but did not differ by day, nor were there any significant interactions between the main effects. Fecal pH was lower in the C–W transition (7.09 ± 0.10) than in the W–C transition (7.42 ± 0.10; *P* = 0.02). Fecal pH was also lower in horses within BRS (6.97 ± 0.13) in comparison to CRS (7.55 ± 0.10; *P* = 0.02). Overall, the lowest recorded fecal pH value was 6.0 for two horses in BRS, one on D2 and the other on D4 of the W–C transition. The highest recorded value for fecal pH was 8.0 for three horses within CRS on various days of both the C–W and the W–C transitions.

## Discussion

Results of this study suggest that the equine microbiome is largely stable during transition between forages within an integrated warm- and cool-season grass rotational grazing system. While species richness did increase by D4 of the transitions and there was a trend for increased phylogenetic diversity (Faith’s Diversity) by D6, evenness and the Shannon Index did not differ over the course of the transitions. Multivariate β-diversity analysis also indicated minimal influence of day in comparison to other variables such as the specific transition between forages (C–W vs. W–C), grazing system (BRS vs. CRS), and individual horse. Additionally, random forest classification was unable to accurately predict days within the C–W and W–C transitions. This was reinforced by the identification of only 3 BCG or ungrouped ASV as differentially abundant across days within specific transitions, with these bacteria representing less than 2% of the total fecal microbial community. Furthermore, fecal pH, as a marker of microbial community function, did not differ by day within transitions between pasture forages. Stability of the microbiota during transitions between pasture forage types indicates that the equine hindgut microbiota is capable of adapting to subtle shifts in nutrient composition between warm- and cool-season grasses.

The lack of differences in these variables across days within transitions may be reflective of the fact that there were only minimal differences in nutritional composition between warm- and cool-season grasses in either system (mean nutrient composition of pasture forages is shown in Table 5). The most pronounced difference in nutrient concentration was the 10% greater crude protein in CRB compared to cool-season grass for the C–W transition in CRS. Water-soluble carbohydrate (WSC; WSC = fructans + sugars) in cool-season grasses were twice that of BER (in BRS) and CRB (in CRS) during the C–W transition. However, the magnitude of these differences was minimal, with WSC concentrations of 5.5 – 7% for cool-season grasses and 3 – 3.5% for BER and CRB. It should be noted that forage processing protocols following sample collection (i.e. forced-air or radiant heat vs. freeze drying) can impact measured plant soluble carbohydrates and that processing samples by forced-air drying as was conducted in the current study would result in lower NSC concentrations due to the continuation of plant respiration through the drying process [33]. However, all samples of all forages in the present study were processed similarly, so this impact of processing method would have been similar in all samples regardless of forage type.

**Table 5:** Nutrient composition^1^ of pasture forages

Nutrients^2^	C–W^3^				W–C^4^			
	BRS^5^		CRS^6^		BRS		CRS	
	CSG	BER	CSG	CRB	BER	CSG	CRB	CSG
Digestible energy, Mcal/kg	2.11	2.12	2.05	2.07	2.17	2.13	2.01	2.17
Crude protein, %	26.3	24.1	13.1	24.6	18.4	17.5	19.0	21.0
Acid detergent fiber, %	31.3	31.1	35.8	34.0	32.2	34.4	38.3	37.0
Neutral detergent fiber, %	58.8	59.3	63.0	60.2	58.7	60.1	63.9	57.6
Non-structural carbohydrates^7^, %	5.1	5.2	6.6	2.9	7.9	7.3	4.5	5.2
Water-soluble carbohydrates %	6.9	3.5	5.5	2.2	5.5	5.9	4.3	3.5
Ethanol-soluble carbohydrates, %	3.1	2.7	3.3	1.8	4.3	5.0	3.8	3.1
Starch, %	0.9	1.7	1.1	0.7	2.4	1.4	2.0	1.7

^1^Nutrient composition of forage samples was determined by near-infrared spectroscopy (Equi-Analytical Laboratories, Ithaca, NY). Concentrations are reported on a dry-matter basis

^2^Nutrient concentrations are reported for hand-clipped representative samples collected in each of the pasture sections on the day of transition

^3^C–W: transitions from cool-season grass (CSG) to warm-season grass

^4^W–C: transitions from warm-season grass to CSG

^5^BRS: bermudagrass (BER) integrated rotational grazing system

^6^CRS: crabgrass (CRB) integrated rotational grazing system

While minimal changes in microbial species composition were observed across days in the current study, differences did occur in BCG containing ASV from the *Lachnospiraceae* family as well as the *NK4A214 group* within *Oscillospiraceae*, *Clostridium *sensu stricto* 1, Anaerovorax,* and *Sarcina.* Differences in *Sarcina* across the W–C transition in CRS were also reflected in the genus-level analysis in addition to fluctuation in *Bacillus* for the W–C in BRS. Garber et al. [34] also found *Sarcina* enriched in horses transitioning to cool-season grass from a hay diet. These results indicate that taxa represented by these ASV, and the *Sarcina* and *Bacillus* genera more broadly, may be more sensitive to subtle changes in pasture forage type and nutrient composition.

Few studies have investigated longitudinal shifts in the hindgut or fecal microbiome of horses during transitions between all-forage diets [34–37], with prior studies primarily focused on abrupt dietary changes between concentrates [38] or abrupt inclusion/elimination of supplemental concentrates in horses maintained on hay or pasture [12, 25, 39, 40]. Garber et al., [34] reported no significant changes in α-diversity (either species richness or evenness) over a 14-d transition between hay and pasture but did identify broad phylum-level changes in microbial composition as well as lower taxa enriched on specific days of the transition periods. In contrast, the current study found changes in species richness, but limited changes in microbial species composition. These differences could be attributable to the fact that Garber et al., [34] evaluated hay and pasture diets, while the current study assessed transition between two pasture forages. To the authors' knowledge, this is the first study to report on transitions between pasture forages or cool- vs. warm-season grasses. The minimal change in microbial species composition in this study is similar to results of previous studies in horses transitioned either between hays [36] or from hay to either haylage or silage of similar botanical composition [41] or between silages varying in crude protein [35].

However, differences in experimental design (i.e. total duration of treatment or adaptation periods, sampling timepoints, etc.) and analytical approaches (culture-based vs. culture-independent, methods of statistical analysis, etc.) make inter-study comparisons difficult. The current study utilized a guild-based analytical approach, grouping individual ASV into BCG prior to random forest classification or analysis of differential abundance, rather than more conventional taxon-based analyses. Given the genetic variation between bacteria within a given taxa (even strains within a single species), members of taxonomic groups may not be functionally homologous and may respond differentially to experimental treatments such as diet or environmental variables [42–45]. Results of this study support this suggestion, as distinct ASV assigned to the same taxonomic lineage were identified in BCG enriched in different transitions, grazing systems, and/or horses. Similarly, Mach et al. [46] reported differential responses of ASV with identical taxonomic assignments in horses transitioned from pasture to a stabled environment for performance training. A number of other recent equine microbiome studies have also evaluated microbial composition at the level of individual ASV [47–49]. Strategies similar to the analyses employed in the current study (grouping bacteria by co-abundance instead of taxonomic rank for purposes of dimensionality reduction) have been previously implemented in studies of the human [50] and mouse gut microbiomes [43, 51, 52]. The use of this BCG-based approach in the current study is further supported by the greater predictive accuracy of random forest classifiers and the greater percentage of the total microbial community captured by differential abundance analysis in comparison to the concurrent taxon-based analysis at the genus level.

Another primary finding of this study was the strong influence of the individual horse on microbial diversity and composition. Analyses of β-diversity metrics by PERMANOVA with Adonis yielded R^2^ values from 0.24 to 0.34, which were 1.5–3.6 times the R^2^ values for the next greatest variable, depending upon the metric analyzed. Application of a random forest classification model indicated that individual horses could be predicted based on microbial community composition with high accuracy, and BCG (and ungrouped ASV) comprising > 32% of the total microbial community composition was identified as differing by horse. Substantial inter-horse variation of the hindgut microbiota has been previously documented in ponies [34, 53] and horses [54–56]. Gomez et al. [48] demonstrated a differential response of individual horses to dietary treatments consisting of a conventional or reduced-lignin alfalfa hay. This individualized response to dietary intervention has also been reported in humans [57] and mice [58].

In the current study, differential abundance analysis found that the most common genera represented in BCG (as well as ungrouped ASV) that differed across horses were *Rikenellaceae RC9 gut group*, *Treponema*, *Christensenellaceae R-7 group*, and the *NK4A214 group* of *Oscillospiraceae*. These genera have all been previously identified in the equine fecal microbiota of forage-fed horses [48, 59, 60] and have been associated with degradation of a diverse array of structural carbohydrates [61–65] as well as production of butyrate and other short-chain fatty acids [66–68]. Interestingly, *Christensenellaceae* has been identified as a highly heritable taxa in the gut of humans and other animal species [61, 69–71], which may explain the inter-individual variability in ASV assigned to this group. Inter-individual variation has also been found in *Treponema*, with Gomez et al. [48] reporting that ASV within this genus displayed differing responses to forage lignin concentration across individual horses. *Rikenellaceae RC9 gut group* was also identified as differentially abundant across horses in the genus-level analysis (in addition to another unclassified genus within *Rikenellaceae*). *Rikenellaceae RC9 gut group* has been identified across a range of herbivorous animal species [72–74] including the horse [75, 76] and has been found to increase in horses transitioned from pasture to a hay-based diet [59]. Members of the *Rikenellaceae* family are specialized to the animal gut [77], and while the role and function of the *RC9 gut group* within the gastrointestinal system has not been fully elucidated [76], this genus has been associated with degradation of structural carbohydrates. Asma et al. [78] found that abundance of *Rikenellaceae RC9 group* decreased as cattle maintained on silage-based diets adapted to supplemental concentrates higher in starch. Members of the *Rikenellaceae* family also degrade mucin, with a documented preference for mucin over simple sugars [78, 79]. It is possible that inter-horse variation in these fiber-degrading genera is indicative of functional redundancy within the hindgut microbial ecosystem and may serve as a mechanism for resilience, allowing horses to withstand subtle changes within the pasture environment.

It should be noted that a limitation of the current study is the relatively small sample size (8 horses). The smaller sample size in this study is similar to sample sizes utilized in prior equine hindgut microbiome studies that have emphasized a strong influence of individual horse [34, 48, 54]. However, it is possible that these small sample sizes are contributing to the strength of the influence of individual horse in comparison to other study variables, and studies in a greater number of horses would provide additional insight regarding any potential impact of transitions between forage types within an integrated grazing system. Additionally, Gomez et al. [35] described individualized responses to dietary intervention, the analysis of which was made possible by twice-daily serial sampling over a 5-d fecal collection period, yielding 10 samples per horse per treatment. In the current study, only one sample was collected on each of 4 days within each transition. While variations in BCG and ungrouped ASV relative abundance were evident in graphical representations of individual horses across days within transitions, additional replications would be required to statistically analyze shifts in bacterial abundances within individual horses. Further research is thus necessary to evaluate whether the microbiota of individual horses are indeed functionally stable across transitions between warm- and cool-season grasses, or to more definitively identify specific bacteria that are shifting in an individualized manner.

Results presented for the current study may have also been impacted by seasonality, as a seasonal control, such as horses maintained on an all-hay diet in which nutrient composition was constant, was not utilized. Seasonal shifts in the gut microbiota have been reported for other non-foraging species including mice [80], monkeys [81], and humans [82], but the effect of seasonality on the equine hindgut microbiome has not been extensively investigated. Theelen et al., [49] reported differences in β-diversity in horses sampled in the summer and winter, regardless of other animal and management variables. In pastured horses assessed monthly over a 12-mon period, Salem et al. [56] found fluctuations in microbial diversity and composition, which were associated with weather variables. This previous study did not evaluate the impact of nutritional composition of the pasture forage. The nutritional profile of pasture forages varies across the grazing season [1, 30, 83] and even over the course of a given day [84–86]. Given the established influence of diet and dietary nutrients on the gut microbiota [26–28] it is likely that changes in pasture forage nutrients also influenced microbial composition of grazing horses evaluated by Salem et al. [56]. Because horses in the current study were rotated through the grazing systems based on forage availability according to rotational grazing best management practices, transitions in each field could not be conducted on the same day. Furthermore, the establishment of BER was delayed in comparison to that of CRB, and thus the timing of productive grazing for these warm-season grasses did not align. However, analysis of individual transitions within each field also did not reveal substantive changes by day.

Additionally, many environmental and management factors can potentially influence nutrient content of pasture forages. Evaluation of the microbiome of horses grazing other cool-season and warm-season grass species and or varieties would be necessary to determine if the stability of the hindgut microbiome found in horses the current study extend to horses grazing in integrated rotational systems containing other forages. Collecting data from multiple years in multiple regional sites would also provide greater insight into the impacts of integrated warm- and cool-season grass rotational grazing systems on the equine hindgut microbial community.

Finally, a complete analysis of microbial functionality also was not conducted in the present study. While limited differences in the structure and composition of the microbiota were observed across days within transitions, it is possible that function could have differed even as bacterial composition remained relatively stable [87]. A number of ASV within the limited BCG varying over days within transitions between cool- and warm-season grasses were assigned to taxa known to degrade fiber as well as other carbohydrates and produce butyrate in addition to other short-chain fatty acids [88–90]. Groups of fibrolytic and butyrate-producing bacteria were differentially abundant across C–W transitions while separate and distinct groups containing ASV assigned to similar taxa and/or taxa with similar ascribed functions varied over the W–C transitions, suggesting functional redundancy and further reinforcing the overall stability of microbial communities during changes in pasture forages. While no differences were found in fecal pH, which is often cited as an indicator of bacterial activity in the hindgut [7, 91, 92], analyses of metabolites such as lactate and short-chain fatty acids or culture-based assays of functional communities (i.e. cellulytics, amylolytics, lactate-utilizers, etc.) would provide a more comprehensive evaluation and deeper understanding of hindgut microbiota function during transitions between warm- and cool-season pasture grasses.

## Conclusions

In conclusion, equine fecal microbial community structure and composition as well as fecal pH are largely stable across transitions between warm-season grass and cool-season grass pasture sections within integrated warm- and cool-season rotational grazing systems. The capacity of the equine hindgut microbiota to adapt to these forages suggests that it is possible to manage healthy adult horses integrated rotational grazing systems of warm- and cool-season grasses without inducing dysfunction, although additional research is necessary to determine if these findings extend to other species and/or varieties of grasses under various environmental and management conditions. The individual horse was the strongest factor influencing the structure and composition of the gut microbiota during transitions, with bacteria representing over 30% of the microbial community differing between horses pointing to possible mechanisms of resilience in response to modest nutrient changes. Fiber-degraders were heavily represented in differentially abundant bacterial groups, regardless of factor (transition, grazing system, or individual horse), but further research is needed to determine if these findings indicate differences in fibrolytic capacity or are reflective of functional redundancy occurring in equine hindgut microbial communities.

### Methods

Research was conducted in 2018 at the Ryders Lane Environmental Best Management Practices Demonstration Horse Farm (Rutgers, The State University of New Jersey; New Brunswick, New Jersey). Weather data for the New Brunswick station nearest to the site was obtained from the Historical Monthly Station Data portal of the Office of the New Jersey State Climatologist website [93]. Monthly average temperatures and precipitation totals across the study period and historical averages can be found in Additional File 6.

### Grazing systems

Two separate 1.5 ha integrated warm- and cool-season rotational grazing systems were utilized: CRS and BRS, as described above. In CRS, 3 sections contained an established cool-season grass mix and the remaining 3 sections were planted with CRB. In BRS, 3 sections contained the established cool-season grass mix and the remaining 3 sections were planted with BER. The cool-season grass mix included *Inavale* Orchardgrass [*Dactylis glomerata* (L.)], *Tower* Tall Fescue (endophyte-free) [*Lolium arundinaceum* (Schreb.) Darbysh.], and *Argyle* Kentucky Bluegrass [*Poa pratensis* (L.)] (DLF Pickseed, Halsey, OR).

### Animal and grazing management

Use of animals in this study was approved by the Rutgers University Institutional Animal Care and Use Committee protocol #PROTO201800013. Eight adult Standardbred mares with a body condition score (BCS; [94]) of 5–7 out of 9 were used for the study. Prior to grazing, horses were weighed and then grouped by age and BW. Groups of four horses were randomly assigned to each of the grazing systems. Initial mean age, BCS, and body weight (BW) for each group is as follows: BRS – 18.0 ± 0.85 yr; 5.6 ± 0.31 (BCS), 549 ± 24 kg; CRS – 17.7 ± 0.44 yr; 5.6 ± 0.13 (BCS), 533 ± 28 kg. Horses received regular veterinary and dental health care administered through Rutgers University Animal Care Program, with the most recent dental examinations conducted within six months prior to the start of the study.

In the spring of 2018 prior to first availability of pasture forage for grazing, each group of horses was housed in a separate dry lot and fed mixed cool-season grass hay at 2.5% BW per day. Horses began grazing on June 7, 2018, when cool-season grass pasture sections reached adequate height for grazing (at least 15.2 cm). Sequential grazing of warm-season grass sections began once the planted forage reached the same 15.2 cm minimum height. In CRS, the CRB was available for first grazing on August 5th. Despite being planted on the same date as CRB, the BER in BRS was slower to establish, with first grazing on September 18th. Weather data across Pastures were managed according to established best management practices for equine rotational grazing [1]. Forage availability dictated rotation frequency, with horses allowed to graze a given pasture section until forage was reduced to approximately 7.6 cm sward height, at which time horses were moved to a new section. After horses were removed from a grazed section, any remaining tall weeds were mowed to a height of 7.6 cm. Pasture sections were then dragged to evenly spread-out manure from defecation areas. Horses grazed on pastures until cool-season grasses became dormant at the end of the growing season in November. When on pasture, horses were allowed 24-h ad libitum access to pasture forage. If adequate pasture forage was not available at any point during the grazing season, horses were confined to a stress lot and supplemental grass hay was provided at 2.5% of body weight (BW) on a dry-matter (DM) basis. Horses had unlimited access to shelters, automatic waterers, and salt blocks throughout the grazing season.

#### Forage sampling

Representative hand-clipped forage samples were collected on the day of rotation. Pasture samples were collected between 0800 and 1000 by walking in a random zig-zag pattern throughout each pasture section, stopping to clip forage (at 7.6 cm height) every 30 paces [1]. Pasture samples were composited and then dried at 60 °C for at least 36 h in a Thelco oven (Precision Scientific, Chicago, IL) and ground to 1 mm using a Wiley Mill. Hay and pasture samples were then submitted to Equi-Analytical Laboratories (Ithaca, NY) to be analyzed by near-infrared spectroscopy.

#### Fecal and sample collection

Manual grab fecal samples were collected rectally from horses during C–W and W–C transitions on the D0, D2, D4, and D6 (see Additional file 7 for a diagram of experimental design and sampling protocol). Prior to the C–W rotation, horses had been grazing within the cool-season pasture sections for a minimum of 21 uninterrupted days (no confinement to stress lots or hay feeding). Similarly, horses grazed the CRB pasture sections in CRS for a minimum of 21 d prior to the W–C transitions. In BRS, only 17 days of grazing BER were possible prior to the W–C transition due to delayed establishment of BER. The duration of grazing prior to transition was selected to represent the longest duration that horses were likely to continuously consume an individual forage type within the context of integrated warm- and cool-season grass rotational grazing management. An adaptation period of 2–3 weeks has been utilized in prior studies evaluating relationships between the diet and the equine microbiome, including in grazing horses, with this period sufficient for stabilization of microbial communities [25, 37, 38]. All samples were collected at 0800 h. Samples were immediately placed on ice and transported from the field to the laboratory (5 min drive). Samples were then stored in a − 80 °C freezer until subsequent analysis.

#### Fecal sample analyses

Fecal pH was measured with a handheld Accumet pH meter (Fisher Scientific; Waltham, MA) calibrated with standard solutions at a pH of 4 and 7. Each sample was analyzed in duplicate, with fecal slurries of 10 mL of dH_2_O and 10 g of fecal matter thoroughly mixed by vortex in two separate 50 mL conical tubes. Two measurements were then collected from each of the duplicate tubes. The pH probe was inserted into the slurry and a reading was collected. The probe was then rinsed with dH_2_O to clean away any fecal material and reinserted to take a second pH reading. Readings were averaged for each horse at each sample point.

DNA was extracted from fecal samples in triplicate using Quick-DNA Fecal/Soil Microbe Kits (Zymo Research; Irvine, CA). Extracted DNA was quantified using a Qubit 2.0 Flourometer (Invitrogen; Carlsbad, CA). For each sample, the highest yielding replicate was selected and submitted to a commercial laboratory for amplification and sequencing of the V4-V5 region of the 16S rRNA gene (RTL Genomics; Lubbock, TX). Region specific primers (515F/926R) were used [95], and sequencing was conducted by Illumina MiSeq. Sequencing blanks were also prepared beginning with the extraction protocol, with sequencing producing zero reads per run for each blank.

#### Sequence and statistical analysis

Sequence and statistical analyses were performed in QIIME 2 (Quantitative Insights Into Microbial Ecology, v. 2020.8) [32] and R (v. 4.0.2) [96]. For all analyses, animal was used as the experimental unit. A record of code utilized in these analyses is included in Additional File 8.

Quality and chimera filtering of paired-end reads was conducted using DADA2 in Qiime 2, with read length set at 260 bp for forward reads and 200 bp for reverse reads [97, 98]. Trees for phylogenetic diversity analyses were generated using the mafft and FastTree pipeline from the q2-phylogeny Qiime 2 plugin [99–101]. Feature tables were filtered so that the lower quartile of ASV based on absolute abundance were removed, with filtering criteria set at a minimum frequency of 9 and presence in a minimum of 4 samples. Bacterial α-diversity and β-diversity analyses were also conducted in Qiime 2, with the feature table rarefied to an even minimum sampling depth of 3700. Differences in α-diversity metrics (Shannon Diversity Index, Faith’s Phylogenetic Diversity, Pielou’s Evenness, and Observed ASVs) were analyzed by mixed model ANOVA in R. System, transition, day, and their interactions were set as fixed factors and transition within horse as the random effect, with means separated by Tukey’s method [102–106]. The β-diversity metrics (Bray–Curtis, Jaccard, Unweighted UniFrac and Weighted Unifrac distance matrices) were analyzed by permutational ANOVA (PERMANOVA) with the Adonis action for multivariate analysis comparisons in Qiime 2 [107–112]. Influence of within-group variance on β-diversity analysis was evaluated by testing homogeneity of dispersion using PERMDISP for individual factors [113].

To further explore differential abundances, ASV were then grouped into BCG based on abundance profiles using SCNIC, with the correlation method set as Spearman and r_s_ ≥ 0.50 [114, 115] with the q2-SCNIC plugin in QIIME 2. Random forest classifiers with nested cross validation (q2-sample-classifier plugin) were applied to determine if metadata variables (grazing system, field, day, horse) could be predicted based on BCG composition [116, 117]. Relative abundances of BCG and ASV that remained ungrouped following application of SCNIC were evaluated by ANCOM [118]. For ANCOM, H_0(*ij*)_: mean(log[x_*i*_/x_*j*_) = mean(log[y_*i*_/y_*j*_). Strength of the ANCOM statistical test is denoted by W values, which indicate the number of times H_0(*ij*)_ is rejected for the *ith* species. For example, if W = 770, the feature was significantly different relative to 770 other features [118]. Taxonomy was assigned to individual ASV using the latest SILVA database (SSU 138) in Qiime 2 [117, 119–122].

Shifts in microbial composition occurring at the genus level were also evaluated by (1.) collapsing ASV by genus, (2.) utilizing SCNIC to group genera by co-abundance, (3.) determining if the variables transition, grazing system, day (for each transition within each grazing system), and horse could be predicted based on microbial composition (random forest classification with nested cross-validation) and (4.) applying ANCOM to identify genera groups that were differentially abundant.

Fecal pH was also analyzed by mixed model ANOVA in R. System, transition, day, and their interactions were set as fixed factors and transition within horse as the random effect, with means separated by Tukey’s method. For α-diversity metrics and pH analyzed by mixed model, normality of model residuals was assessed using the Shapiro–Wilk test, and means were separated using Tukey’s method. Data for variables analyzed by mixed model are presented as means ± SEM. For all analyses which generated *P*-values, results were considered significant at *P* ≤ 0.05, with trends considered at *P* ≤ 0.10.

## Supplementary Information

**Additional file 1: **Taxonomic classification of amplicon sequence variants (ASV) retained after quality filtering, chimera checking, and filtering low-abundance ASV.

**Additional file 2:** Differentially abundant bacterial co-abundance groups (BCG) and ungrouped amplicon sequence variants (ASV) across horses.

**Additional file 3:** Relative abundance of the twelve most abundant BCG that differed by horse.

**Additional file 4:** Genera groups and/or ungrouped genera identified as differentially abundant by grazing system, transition, day (within each grazing system for each transition) and horse.

**Additional file 5:** Analysis comparison of grouping amplicon sequence variants into bacterial co-abundance groups (BCG) versus genus-level groupings.

**Additional file 6:** Monthly average temperatures and precipitation totals across the study period and historical averages.

**Additional file 7:** Diagram of experimental design and sampling protocol.

**Additional file 8:** Code used for sequence and statistical analysis in Qiime 2 (v.2020.8) and R (v. 4.0.2).

## Data Availability

The datasets generated and analyzed during the current study are available in the NCBI Sequence Read Archive at https://www.ncbi.nlm.nih.gov/sra, Bioproject: PRJNA804247, Accession numbers: SAMN25718984- SAMN25719047.

## Abbreviations

ANCOM: Analysis of Composition of Microbes
ASV: Amplicon sequence variants
BER: *Wrangler* Bermudagrass
BCG: Bacterial co-abundance groups
BCS: Body condition score
BRS: Bermudagrass and cool-season grass integrated rotational grazing system
BW: Body weight
CRB: *Quick-N-Big* Crabgrass
CRS: Crabgrass and cool-season grass integrated rotational grazing system
C–W: Cool-season grass to warm-season grass transition
D0: Day zero of rotation
D2: Day two of rotation
D4: Day four of rotation
D6: Day six of rotation
DM: Dry-matter
NSC: Non-structural carbohydrates
PERMANOVA: Permutational analysis of variance
PERMDISP: Permutational analysis of dispersion
SCNIC: Sparce Cooccurence Network Investigation for Compositional Data
WSC: Water-soluble carbohydrates
W–C: Warm-season grass to cool-season grass transition
