# Supplementary material for: Fecal microbiome of horses transitioning between warm-season and cool-season grass pasture within integrated rotational grazing systems
Source: Anim Microbiome. 2022 Jun 21;4:41. doi: 10.1186/s42523-022-00192-x (PMC9210719; doi:10.1186/s42523-022-00192-x)
Supplement: Supplementary file 1 — Additional file 1: Taxonomic classification of amplicon sequence variants (ASV) retained after quality filtering, chimera checking, and filtering low-abundance ASV. [file 42523_2022_192_MOESM1_ESM.pdf]

Additional File 1. Taxonomic classification[1] of all amplicon sequence variants (ASV)[2].

Taxonomic Lineage							ASV Count[3]
**Kingdom**	**Phylum**	**Class**	**Order**	**Family**	**Genus**	**Species**	
Archaea	*Euryarchaeota*	*Methanobacteria*	*Methanobacteriales*	*Methanobacteriaceae*	*Methanobrevibacter*	-------------	1
						M. gottschalkii	1
						M. ruminatium	2
	Halobacterota	*Methanomicrobia*	*Methanomicrobiales*	*Methanocorpusculaceae*	*Methanocorpusculum*	-------------	5
	Thermoplasmatota	*Thermoplasmata*	*Methanomassiliicoccales*	*Methanomethylophilaceae*	*uncultured*	*uncultured rumen*	1
Bacteria	-------------	-------------	-------------	-------------	-------------	-------------	10
Bacteria	*Actinobacteriota*	*Coriobacteriia*	*Coriobacteriales*	-------------	-------------	-------------	3
				Coriobacteriales	*Coriobacteriales*	*uncultured bacterium*	2
				Coriobacteriales Incertae Sedis	*Phoenicibacteer*	*uncultured bacterium*	2
					uncultured	-------------	1
				Eggerthellaceae	-------------	-------------	5
					Denitrobacterium	*D. detoxificans*	1
Bacteria	*Armatimonadota*	*Chthonomonadetes*	*Chthonomonadales*	*Chthonomonadales*	*Chthonomonadales*	*uncultured Lutispora*	1
Bacteria	*Bacteroidetes*	*Bacteroidia*	-------------	-------------	-------------	-------------	18
			Bacteroidales	-------------	-------------	-------------	26
				Bacteroidaceae	*Bacteroides*	-------------	6
				Bacteroidales BS11 gut group	*Bacteroidales BS11 gut group*	*uncultured Bacteroidetes*	3
				Bacteroidales RF16 group	*Bacteroidales RF16 group*	-------------	4
						rumen bacterium	1
						uncultured bacterium	1
						uncultured Paludibacter	4
				Bacteroidales UCG-001	*Bacteroidales UCG-001*	-------------	8
						uncultured bacterium	4
						uncultured Bacteroidales	1
				Bacteroidetes BD2-2	*Bacteroidetes BD2-2*	*uncultured rumen*	1
				COB P4-1 termite group	*COB P4-1 termite group*	*uncultured rumen*	1

F082	*F082*	-------------	2
		bacterium P201	9
		Bacteroidales bacterium	5
		Bacteroidia bacterium	6
gir-aah93h0	*gir-aah93h0*	*uncultured bacterium*	1
LKC2 127-25	*LKC2 127-25*	*uncultured Bacteroidetes*	1
M2PB4-65 termite group	*M2PB4-65 termite group*	-------------	2
		metagenome	1
		uncultured Bacteroidetes	2
Marinifilaceae	*uncultured*	*uncultured rumen*	1
Muribaculaceae	*Muribaculaceae*	-------------	1
		uncultured rumen	1
		uncultured bacterium	2
p-251-o5	*p-251-o5*	-------------	1
		uncultured bacterium	14
Paludibacteraceae	*uncultured*	-------------	1
Prevotellaceae	-------------	-------------	12
	Alloprevotella	-------------	3
		Prevotellaceae bacterium	1
	Prevotella	-------------	10
	Prevotellaceae Ga6A1 group	-------------	3
		rumen bacterium	1
		unidentified rumen	5
	Prevotellaceae UCG-001	-------------	1
		uncultured bacterium	2
		uncultured Prevotellaceae	1
		wallaby gut	6
	Prevotellaceae UCG-003	-------------	3

						Alloprevotella sp.	4
						uncultured Bacteroidales	1
				Rikenellaceae	-------------	-------------	5
					Alistipes	-------------	1
					hoa5-07d05 gut group	*uncultured bacterium*	11
					Mucinivorans	*uncultured bacterium*	1
					Rikenellaceae RC9 gut group	-------------	25
						uncultured beta	9
						uncultured prokaryote	2
						uncultured Rikenella	29
						wallaby gut	5
					SP3-e08	*uncultured bacterium*	7
Bacteria	*Chloroflexi*	*Anaerolineae*	*Anaerolineales*	*Anaerolineaceae*	*Flexilinea*	*F. flocculi*	1
Bacteria	*Desulfobacterota*	*Desulfovibrionia*	*Desulfovibrionales*	*Desulfovibrionaceae*	*Desulfovibrio*	-------------	2
					Mailhella	*uncultured bacterium*	1
Bacteria	*Fibrobacterota*	*Fibrobacteria*	*Fibrobacterales*	*Fibrobacteraceae*	*Fibrobacter*	-------------	3
						bacterium MB2022	1
						uncultured Fibrobacteraceae	1
Bacteria	*Firmicutes*	-------------	-------------	-------------	-------------	-------------	5
		Bacilli	-------------	-------------	-------------	-------------	1
			Bacillales	*Bacillaceae*	*Bacillus*	-------------	1
			Erysipelotrichales	*Erysipelatoclostridiaceae*	-------------	-------------	1
					Erysipelatoclostridium	-------------	2
					UCG-004	-------------	6
						uncultured rumen	1
					uncultured	*uncultured Erysipelotrichaceae*	1
				Erysipelotrichaceae	-------------	-------------	2
					Catenisphaera	*uncultured bacterium*	1
					Holdemania	-------------	1
					uncultured	*uncultured bacterium*	3

	Lactobacillales	*Lactobacillaceae*	*Lactobacillus*	*L. equigenerosi*	1
				L. hayakitensis	1
	Mycoplasmatales	*Mycoplasmataceae*	*Mycoplasma*	-------------	1
	RF39	*RF39*	*RF39*	-------------	12
				anaerobic digester Firmicutes bacterium	1
				uncultured Lachnospiraceae	1
				unidentified rumen	1
Clostridia	-------------	-------------	-------------	-------------	11
	Christensenellales	*Christensenellaceae*	*Christensenellaceae R-7 group*	-------------	17
				bacterium AC043	32
				bacterium YE57	1
				Christensenella sp.	1
				uncultured Christensenella	14
				uncultured Clostridia	1
				uncultured Lachnospiraceae	5
				uncultured prokaryote	2
	Clostridia	*Hungateiclostridiaceae*	-------------	-------------	1
			Saccharofermentans	-------------	6
				Ruminococcaceae bacterium	1
	Clostridia UCG-014	*Clostridia UCG-014*	*Clostridia UCG-014*	-------------	3
				uncultured Lactococcus	1
	Clostridiales	*Clostridiaceae*	*Clostridium sensu stricto 1*	-------------	7
				C. chartatabidum	1
			Sarcina	*S. maxima*	2
	Eubacteriales	*Anaerofustaceae*	*Anaerofustis*	-------------	1
		Eubacteriaceae	*Eubacterium*	-------------	4
				uncultured bacterium	2
	Lachnospirales	*Defluviitaleaceae*	*Defluviitaleaceae UCG-011*	*uncultured Eubacteriaceae*	1

	Lachnospiraceae	-------------	-------------	133
		[Eubacterium]hallii group	-------------	3
			Clostridium sp.	2
			uncultured Clostridia	1
			uncultured Lachnospiraceae	1
		[Eubacterium] ruminantium group	*bacterium YE64*	1
		Acetitomaculum	*uncultured bacterium*	3
		Agathobacter	*uncultured rumen*	1
		Blautia	-------------	6
		Coprococcus	-------------	1
		Frisingicoccus	*uncultured Firmicutes*	2
		Lachnoclostridium	-------------	5
			[Clostridium asparagiforme]	1
		Lachnospiraceae AC2044 group	-------------	1
		Lachnospiraceae FE2018 group	-------------	1
		Lachnospiraceae ND3007 group	*uncultured Lachnospiraceae*	2
		Lachnospiraceae UCG-002	*uncultured Lachnospiraceae*	1
		Lachnospiraceae UCG-009	*uncultured Lachnospiraceae*	2
		Lachnospiraceae XPB1014 group	-------------	1
		Marvinbryantia	-------------	1
			uncultured Lachnospiraceae	1
			wallaby gut	5
		Pseudobutyrivibrio	-------------	2
		uncultured	*uncultured Pseudobutyvibrio*	1
Monoglobales	*Monoglobaceae*	*Monoglobus*	-------------	2
Oscillospirales	*[Eubacterium] coprostanoligenes group*	*[Eubacterium] coprostanoligenes group*	-------------	26
			human gut	6

		metagenome	1
		uncultured Clostridia	3
		uncultured Clostridiales	2
		uncultured Clostridium	6
		uncultured Eubacterium	1
		uncultured prokaryote	1
		uncultured Ruminococcaceae	4
Butyricicoccaceae	*UCG-009*	-------------	5
Ethanoligenenaceae	-------------	-------------	1
Ethanoligenenaceae	*Ethanoligenens*	*E. harbinense*	1
Oscillospiraceae	-------------	-------------	15
	Colidextribacter	-------------	1
	Intestinimonas	*I. timonensis*	2
	NK4A214 group	-------------	11
		gut metagenome	3
		metagenome	3
		uncultured Lachnospiraceae	1
		uncultured rumen	17
	Sporobacter	-------------	3
	UCG-002	-------------	5
		uncultured Ruminococcaceae	6
	UCG-005	-------------	9
		human gut	5
		metagenome	1
		unidentified	4
	uncultured	*metagenome*	1
Ruminococcaceae	-------------	-------------	6
	Anaerofilum	-------------	1
	Candidatus Soleaferrea	-------------	1
	Fournierella	*Uncultured Firmicutes*	1
	Insertae Sedis	-------------	1

				Ruminococcus	-------------	3
					bacterium FB2012	6
					Bacteroidetes bacterium	3
					R. champanellensis	1
				uncultured	*uncultured Clostridium*	3
			UCG-010	*UCG-010*	-------------	24
					bacterium enrichment	1
					metagenome	3
					uncultured Bacillus	1
					uncultured Clostridia	9
					uncultured eubacterium	1
					uncultured organism	7
					unidentified	2
					unidentified rumen	1
		Peptostreptococcales-Tissierellales	*Anaerovoracaceae*	-------------	-------------	6
				[Eubacterium] nodatum group	-------------	2
				Anaerovorax	-------------	5
					Peptostreptococcaceae bacterium	2
					uncultured rumen	2
				Family XIII AD3011 group	-------------	2
					bacterium AD3011	4
					gut metagenome	2
					uncultured Clostridiales	1
				Family XIII UCG-001	-------------	1
					uncultured rumen	2
				Mogibacterium	-------------	3
					uncultured rumen	3
			Peptostreptococcaceae	*Terrisporobacter*	-------------	1
	Negativicutes	*Acidaminococcales*	*Acidaminococcaceae*	*Phascolarctobacterium*	-------------	8
					P. succinatutens	1

						wallaby gut	2
			Veillonellales-Selenomonadales	*Selenomonadaceae*	-------------	-------------	1
					Schwartzia	-------------	1
Bacteria	*Planctomycetota*	*Planctomycetes*	*Pirellulales*	*Pirellulaceae*	*p-1088-a5 gut group*	*uncultured bacterium*	7
						uncultured rumen	2
		vadinHA49	*vadinHA49*	*vadinHA49*	*vadinHA49*	*uncultured Planctomycetaceae*	1
						uncultured sediment	1
Bacteria	*Proteobacteria*	*Alphaproteobacteria*	*Rhodospirillales*	*uncultured*	*uncultured*	*uncultured rumen*	1
		Gammaproteobacteria	*Burkholderiales*	-------------	-------------	-------------	1
				Oxalobacteraceae	-------------	-------------	1
				T34	*T34*	*uncultured bacterium*	1
Bacteria	*Spirochaetota*	*MVP-15*	*MVP-15*	*MVP-15*	*MVP-15*	-------------	1
						uncultured rumen	1
		Spirochaetia	*Spirochaetales*	*Spirochaetaceae*	*Sphaerochaeta*	-------------	1
						uncultured rumen	4
						uncultured rumen	7
					Treponema	-------------	24
						bacterium MD2012	1
						T. brennaborense	1
						T. porcinum	9
						T. ruminis	1
Bacteria	*Synergistota*	*Synergistia*	*Synergistales*	*Synergistaceae*	-------------	-------------	2
					uncultured	*uncultured bacterium*	6
Bacteria	*Verrucomicrobiota*	*Kiritimatiellae*	*WCHB1-41*	*WCHB1-41*	*WCHB1-41*	-------------	6
						uncultured bacterium	7
						uncultured prokaryote	2
						uncultured rumen	7
		Verrucomicrobiae	*LD1-PB3*	*LD1-PB3*	*LD1-PB3*	*uncultured rumen*	1
			Pedosphaerales	*Pedosphaeraceae*	*DEV114*	-------------	1
						uncultured rumen	1
			Verrucomicrobiales	*Akkermansiaceae*	*Akkermansia*	-------------	3

Verrucomicrobiales	*Akkermansiaceae*	*Akkermansia*	*A. glycaniphila*	1

[1] Taxonomic assignment was conducted in Qiime 2 using the most recent SILVA database (SSU 138).

[2] The ASV represent those retained after quality filtering, chimera checking, and filtering low abundance ASV, but prior to rarefaction for subsequent diversity analyses.

[3] Counts of individual ASV with a shared lineage.
