# Supplementary material for: Fecal microbiome of horses transitioning between warm-season and cool-season grass pasture within integrated rotational grazing systems
Source: Anim Microbiome. 2022 Jun 21;4:41. doi: 10.1186/s42523-022-00192-x (PMC9210719; doi:10.1186/s42523-022-00192-x)
Supplement: Supplementary file 2 — Additional file 2: Differentially abundant bacterial co-abundance groups (BCG) and ungrouped amplicon sequence variants (ASV) across horses. [file 42523_2022_192_MOESM2_ESM.pdf]

Additional File 3. Differentially abundant[1] bacterial co-abundance groups (BCG)[2] and ungrouped amplicon sequence variants (ASV) across horses.

BCG	W [3]	Taxonomic Lineage [4]
**BCG_0**	**771**	*p__Firmicutes; c__Negativicutes; o__Acidaminococcales; f__Acidaminococcaceae; g__Phascolarctobacterium*
		p__Bacteroidota; c__Bacteroidia; o__Bacteroidales
		p__Firmicutes; c__Clostridia; o__Peptostreptococcales-Tissierellales; f__Anaerovoracaceae; g__Mogibacterium; s__uncultured_rumen
		p__Firmicutes; c__Clostridia; o__Oscillospirales; f__Oscillospiraceae; g__NK4A214_group; s__unidentified_rumen
		p__Bacteroidota; c__Bacteroidia; o__Bacteroidales; f__p-251-o5; g__p-251-o5
		p__Bacteroidota; c__Bacteroidia; o__Bacteroidales; f__Prevotellaceae; g__Prevotellaceae_Ga6A1_group; s__unidentified_rumen
		p__Bacteroidota; c__Bacteroidia; o__Bacteroidales; f__Bacteroidales_UCG-001; g__Bacteroidales_UCG-001
		p__Firmicutes; c__Clostridia; o__Christensenellales; f__Christensenellaceae ; g__Christensenellaceae _R-7_group; s__bacterium_AC2043
		p__Firmicutes; c__Clostridia; o__Christensenellales; f__Christensenellaceae ; g__Christensenellaceae _R-7_group; s__bacterium_AC2043
		p__Bacteroidota; c__Bacteroidia; o__Bacteroidales; f__Rikenellaceae; g__Rikenellaceae_RC9_gut_group
		p__Firmicutes; c__Clostridia; o__Lachnospirales; f__Lachnospiraceae
		p__Firmicutes; c__Clostridia; o__Oscillospirales; f__Oscillospiraceae; g__NK4A214_group; s__metagenome
		p__Planctomycetota; c__Planctomycetes; o__Pirellulales; f__Pirellulaceae; g__p-1088-a5_gut_group; s__uncultured_bacterium
**BCG_1**	**771**	*p__Bacteroidota; c__Bacteroidia; o__Bacteroidales; f__Rikenellaceae; g__Rikenellaceae_RC9_gut_group*
		p__Firmicutes; c__Clostridia; o__Lachnospirales; f__Lachnospiraceae
		p__Bacteroidota; c__Bacteroidia; o__Bacteroidales; f__Prevotellaceae
		p__Bacteroidota; c__Bacteroidia; o__Bacteroidales
		p__Bacteroidota; c__Bacteroidia; o__Bacteroidales; f__Rikenellaceae; g__Rikenellaceae_RC9_gut_group
		p__Bacteroidota; c__Bacteroidia; o__Bacteroidales; f__F082; g__F082; s__bacterium_P201
		p__Bacteroidota; c__Bacteroidia
		p__Bacteroidota; c__Bacteroidia; o__Bacteroidales; f__F082; g__F082; s__bacterium_P201
		p__Bacteroidota; c__Bacteroidia; o__Bacteroidales
		p__Bacteroidota; c__Bacteroidia; o__Bacteroidales
**BCG_2**	**770**	*p__Bacteroidota; c__Bacteroidia; o__Bacteroidales; f__Rikenellaceae*
		p__Bacteroidota; c__Bacteroidia; o__Bacteroidales; f__Rikenellaceae; g__Rikenellaceae_RC9_gut_group; s__uncultured_beta
		p__Halobacterota; c__Methanomicrobia; o__Methanomicrobiales; f__Methanocorpusculaceae; g__Methanocorpusculum
		p__Firmicutes; c__Clostridia; o__Lachnospirales; f__Lachnospiraceae; g__Lachnospiraceae_XPB1014_group
		p__Bacteroidota; c__Bacteroidia; o__Bacteroidales; f__F082; g__F082; s__bacterium_P201
		p__Bacteroidota; c__Bacteroidia; o__Bacteroidales; f__F082; g__F082; s__Bacteroidales_bacterium
		p__Firmicutes; c__Clostridia; o__Oscillospirales; f__Butyricicoccaceae; g__UCG-009
		p__Halobacterota; c__Methanomicrobia; o__Methanomicrobiales; f__Methanocorpusculaceae; g__Methanocorpusculum
		p__Bacteroidota; c__Bacteroidia; o__Bacteroidales; f__Rikenellaceae; g__Rikenellaceae_RC9_gut_group

**BCG_3**	**770**	*p__Bacteroidota; c__Bacteroidia; o__Bacteroidales; f__Rikenellaceae; g__Alistipes*<br>*p__Bacteroidota; c__Bacteroidia; o__Bacteroidales; f__Rikenellaceae; g__Rikenellaceae_RC9_gut_group*<br>*p__Bacteroidota; c__Bacteroidia; o__Bacteroidales; f__Rikenellaceae; g__Rikenellaceae_RC9_gut_group; s__uncultured_beta*<br>*p__Bacteroidota; c__Bacteroidia; o__Bacteroidales; f__F082; g__F082; s__bacterium_P201*<br>*p__Verrucomicrobiota; c__Kiritimatiellae; o__WCHB1-41; f__WCHB1-41; g__WCHB1-41; s__uncultured_bacterium*<br>*p__Bacteroidota; c__Bacteroidia; o__Bacteroidales; f__Rikenellaceae; g__Rikenellaceae_RC9_gut_group*<br>*p__Bacteroidota; c__Bacteroidia; o__Bacteroidales; f__Rikenellaceae; g__Rikenellaceae_RC9_gut_group*<br>*p__Bacteroidota; c__Bacteroidia; o__Bacteroidales; f__Prevotellaceae*<br>*p__Firmicutes; c__Clostridia; o__Oscillospirales*
**BCG_6**	**771**	*p__Firmicutes; c__Clostridia; o__Christensenellales; f__Christensenellaceae ; g__Christensenellaceae _R-7_group; s__uncultured_Lachnospiraceae*<br>*p__Firmicutes; c__Clostridia; o__Christensenellales; f__Christensenellaceae ; g__Christensenellaceae _R-7_group; s__uncultured_Christensenella*<br>*p__Firmicutes; c__Clostridia; o__Christensenellales; f__Christensenellaceae ; g__Christensenellaceae _R-7_group; s__uncultured_Christensenella*<br>*p__Firmicutes; c__Clostridia; o__Peptostreptococcales-Tissierellales; f__Anaerovoracaceae; g__Family_XIII_UCG-001; s__uncultured_rumen*<br>*p__Firmicutes; c__Clostridia; o__Oscillospirales; f__UCG-010; g__UCG-010; s__uncultured_Clostridia*<br>*p__Spirochaetota; c__Spirochaetia; o__Spirochaetales; f__Spirochaetaceae; g__Treponema*
**BCG_7**	**771**	*p__Firmicutes; c__Clostridia; o__Christensenellales; f__Christensenellaceae ; g__Christensenellaceae _R-7_group; s__uncultured_Christensenella*<br>*p__Firmicutes; c__Clostridia; o__Christensenellales; f__Christensenellaceae ; g__Christensenellaceae _R-7_group; s__uncultured_Christensenella*<br>*p__Spirochaetota; c__Spirochaetia; o__Spirochaetales; f__Spirochaetaceae; g__Treponema*<br>*p__Bacteroidota; c__Bacteroidia; o__Bacteroidales; f__Prevotellaceae; g__Prevotellaceae_Ga6A1_group; s__unidentified_rumen*<br>*p__Firmicutes; c__Clostridia; o__Christensenellales; f__Christensenellaceae ; g__Christensenellaceae _R-7_gro*
**BCG_9**	**762**	*p__Firmicutes; c__Clostridia; o__Oscillospirales; f__Oscillospiraceae; g__UCG-002*<br>*p__Firmicutes; c__Clostridia; o__Oscillospirales; f__Oscillospiraceae*<br>*p__Bacteroidota; c__Bacteroidia; o__Bacteroidales; f__F082; g__F082; s__Bacteroidales_bacterium*<br>*p__Bacteroidota; c__Bacteroidia; o__Bacteroidales; f__Rikenellaceae; g__Rikenellaceae_RC9_gut_group; s__uncultured_Rikenella*<br>*p__Bacteroidota; c__Bacteroidia; o__Bacteroidales; f__Bacteroidales_UCG-001; g__Bacteroidales_UCG-001*
**BCG_14**	**764**	*p__Bacteroidota; c__Bacteroidia; o__Bacteroidales; f__Bacteroidales_UCG-001; g__Bacteroidales_UCG-001; s__uncultured_bacterium*<br>*p__Firmicutes; c__Clostridia; o__Oscillospirales; f__UCG-010; g__UCG-010*<br>*p__Bacteroidota; c__Bacteroidia; o__Bacteroidales; f__Rikenellaceae; g__Rikenellaceae_RC9_gut_group; s__uncultured_prokaryote*<br>*p__Bacteroidota; c__Bacteroidia; o__Bacteroidales; f__Prevotellaceae*
**BCG_15**	**767**	*p__Firmicutes; c__Clostridia; o__Peptostreptococcales-Tissierellales; f__Anaerovoracaceae*<br>*p__Bacteroidota; c__Bacteroidia; o__Bacteroidales; f__Rikenellaceae; g__Rikenellaceae_RC9_gut_group*<br>*p__Firmicutes; c__Clostridia; o__Peptostreptococcales-Tissierellales; f__Anaerovoracaceae*<br>*p__Spirochaetota; c__Spirochaetia; o__Spirochaetales; f__Spirochaetaceae; g__Treponema ; s__bacterium_MD2012*
**BCG_20**	**770**	*p__Bacteroidota; c__Bacteroidia*<br>*p__Bacteroidota; c__Bacteroidia; o__Bacteroidales; f__Prevotellaceae; g__Prevotellaceae_UCG-001; s__wallaby_gut*<br>*p__Spirochaetota; c__Spirochaetia; o__Spirochaetales; f__Spirochaetaceae; g__Treponema*

**BCG_21**	**771**	*p__Firmicutes; c__Clostridia; o__Oscillospirales; f__[Eubacterium]_coprostanoligenes_group; g__[Eubacterium]_coprostanoligenes_group*
		p__Firmicutes; c__Clostridia; o__Oscillospirales; f__Oscillospiraceae; g__NK4A214_group; s__unidentified_rumen
		p__Firmicutes; c__Clostridia; o__Oscillospirales; f__UCG-010; g__UCG-010; s__uncultured_eubacterium
**BCG_22**	**771**	*p__Firmicutes; c__Bacilli; o__Erysipelotrichales; f__ErysipelatoClostridiaceae; g__Erysipelatoclostridium*
		p__Firmicutes; c__Clostridia; o__Lachnospirales; f__Lachnospiraceae
		p__Bacteroidota; c__Bacteroidia; o__Bacteroidales; f__Prevotellaceae
**BCG_25**	**768**	*p__Bacteroidota; c__Bacteroidia; o__Bacteroidales; f__Prevotellaceae*
		p__Firmicutes; c__Clostridia; o__Lachnospirales; f__Lachnospiraceae; g__Lachnospiraceae_UCG-009; s__uncultured_Lachnospiraceae
		p__Bacteroidota; c__Bacteroidia; o__Bacteroidales
**BCG_26**	**771**	*p__Bacteroidota; c__Bacteroidia*
		p__Bacteroidota; c__Bacteroidia; o__Bacteroidales; f__Rikenellaceae; g__Rikenellaceae_RC9_gut_group; s__uncultured_Rikenella
		p__Bacteroidota; c__Bacteroidia; o__Bacteroidales; f__Rikenellaceae; g__Rikenellaceae_RC9_gut_group; s__uncultured_Rikenella
**BCG_28**	**761**	*p__Bacteroidota; c__Bacteroidia; o__Bacteroidales*
		p__Bacteroidota; c__Bacteroidia; o__Bacteroidales
		p__Spirochaetota; c__MVP-15; o__MVP-15; f__MVP-15; g__MVP-15
**BCG_31**	**771**	*p__Firmicutes; c__Clostridia; o__Clostridia; f__HungateiClostridiaceae; g__Saccharofermentans*
		p__Firmicutes; c__Clostridia; o__Lachnospirales; f__Lachnospiraceae
		p__Bacteroidota; c__Bacteroidia; o__Bacteroidales; f__Bacteroidales_UCG-001; g__Bacteroidales_UCG-001
**BCG_32**	**722**	*p__Firmicutes; c__Clostridia; o__Oscillospirales; f__Ruminococcaceae*
		p__Halobacterota; c__Methanomicrobia; o__Methanomicrobiales; f__Methanocorpusculaceae; g__Methanocorpusculum
		p__Bacteroidota; c__Bacteroidia; o__Bacteroidales; f__Bacteroidaceae; g__Bacteroides
**BCG_34**	**762**	*p__Bacteroidota; c__Bacteroidia; o__Bacteroidales; f__Rikenellaceae; g__Rikenellaceae_RC9_gut_group*
		p__Bacteroidota; c__Bacteroidia; o__Bacteroidales; f__Rikenellaceae; g__Rikenellaceae_RC9_gut_group
		p__Firmicutes; c__Clostridia; o__Christensenellales; f__Christensenellaceae ; g__Christensenellaceae _R-7_group; s__bacterium_AC2043
**BCG_36**	**769**	*p__Synergistota; c__Synergistia; o__Synergistales; f__Synergistaceae; g__uncultured; s__uncultured_rumen*
		p__Firmicutes; c__Clostridia; o__Oscillospirales; f__Oscillospiraceae; g__NK4A214_group; s__unidentified_rumen
		p__Planctomycetota; c__Planctomycetes; o__Pirellulales; f__Pirellulaceae; g__p-1088-a5_gut_group; s__uncultured_bacterium
**BCG_43**	**769**	*p__Bacteroidota; c__Bacteroidia; o__Bacteroidales; f__Prevotellaceae; g__Prevotellaceae_Ga6A1_group; s__unidentified_rumen*
		p__Fibrobacterota; c__Fibrobacteria; o__Fibrobacterales; f__Fibrobacteraceae; g__Fibrobacter; s__bacterium_MB2022
		p__Bacteroidota; c__Bacteroidia; o__Bacteroidales; f__Prevotellaceae; g__Prevotellaceae_Ga6A1_group; s__unidentified_rumen
**BCG_45**	**748**	*p__Spirochaetota; c__Spirochaetia; o__Spirochaetales; f__Spirochaetaceae; g__Treponema*
		p__Firmicutes; c__Clostridia; o__Lachnospirales; f__Lachnospiraceae
		p__Spirochaetota; c__Spirochaetia; o__Spirochaetales; f__Spirochaetaceae; g__Treponema

**BCG_47**	**726**	*p__Firmicutes; c__Clostridia; o__Oscillospirales; f__Oscillospiraceae; g__NK4A214_group; s__uncultured_Lachnospiraceae*<br>*p__Firmicutes; c__Clostridia; o__Oscillospirales; f__UCG-010; g__UCG-010*<br>*p__Firmicutes; c__Clostridia; o__Oscillospirales; f__Oscillospiraceae; g__NK4A214_group; s__uncultured_Lachnospiraceae*
**BCG_49**	**771**	*p__Firmicutes; c__Clostridia; o__Oscillospirales; f__Oscillospiraceae; g__UCG-002*<br>*p__Bacteroidota; c__Bacteroidia; o__Bacteroidales; f__Bacteroidales_BS11_gut_group; g__Bacteroidales_BS11_gut_group; s__uncultured_Bacteroidetes*<br>*p__Bacteroidota; c__Bacteroidia; o__Bacteroidales; f__Rikenellaceae; g__SP3-e08; s__uncultured_bacterium*
**BCG_51**	**771**	*p__Bacteroidota; c__Bacteroidia; o__Bacteroidales; f__Rikenellaceae; g__Rikenellaceae_RC9_gut_group; s__uncultured_Rikenella*<br>*p__Bacteroidota; c__Bacteroidia; o__Bacteroidales; f__Marinifilaceae; g__uncultured; s__uncultured_rumen*<br>*p__Bacteroidota; c__Bacteroidia; o__Bacteroidales; f__Rikenellaceae; g__Rikenellaceae_RC9_gut_group; s__uncultured_Rikenella*
**BCG_66**	**764**	*p__Bacteroidota; c__Bacteroidia; o__Bacteroidales; f__Prevotellaceae; g__Prevotella*<br>*p__Bacteroidota; c__Bacteroidia; o__Bacteroidales; f__Prevotellaceae; g__Prevotella*
**BCG_69**	**704**	*p__Bacteroidota; c__Bacteroidia; o__Bacteroidales; f__Rikenellaceae; g__SP3-e08; s__uncultured_bacterium*<br>*p__Spirochaetota; c__Spirochaetia; o__Spirochaetales; f__Spirochaetaceae; g__Treponema ; s__Treponema _porcinum*
**BCG_73**	**770**	*p__Firmicutes; c__Bacilli; o__Erysipelotrichales; f__ErysipelatoClostridiaceae; g__UCG-004*<br>*p__Bacteroidota; c__Bacteroidia; o__Bacteroidales; f__Rikenellaceae*
**BCG_82**	**750**	*p__Desulfobacterota; c__Desulfovibrionia; o__Desulfovibrionales; f__Desulfovibrionaceae; g__Desulfovibrio*<br>*p__Actinobacteriota; c__Coriobacteriia; o__Coriobacteriales; f__Eggerthellaceae; g__Denitrobacterium; s__Denitrobacterium_detoxificans*
**BCG_98**	**768**	*p__Firmicutes; c__Clostridia; o__Peptostreptococcales-Tissierellales; f__Anaerovoracaceae; g__Mogibacterium*<br>*p__Firmicutes; c__Clostridia; o__Oscillospirales; f__Oscillospiraceae; g__NK4A214_group*
**BCG_101**	**754**	*p__Bacteroidota; c__Bacteroidia; o__Bacteroidales; f__Prevotellaceae; g__Prevotella*<br>*p__Firmicutes; c__Clostridia; o__Oscillospirales*
**BCG_102**	**711**	*p__Spirochaetota; c__Spirochaetia; o__Spirochaetales; f__Spirochaetaceae; g__Treponema ; s__Treponema _porcinum*<br>*p__Firmicutes; c__Clostridia; o__Oscillospirales; f__Oscillospiraceae; g__NK4A214_group; s__unidentified_rumen*
**BCG_104**	**771**	*p__Bacteroidota; c__Bacteroidia; o__Bacteroidales*<br>*p__Bacteroidota; c__Bacteroidia*
**BCG_128**	**756**	*p__Firmicutes; c__Clostridia; o__Oscillospirales; f__[Eubacterium]_coprostanoligenes_group; g__[Eubacterium]_coprostanoligenes_group; s__uncultured_Clostridiales*<br>*p__Spirochaetota; c__Spirochaetia; o__Spirochaetales; f__Spirochaetaceae; g__Treponema*
**BCG_135**	**770**	*p__Spirochaetota; c__Spirochaetia; o__Spirochaetales; f__Spirochaetaceae; g__Treponema ; s__Treponema _porcinum*<br>*p__Firmicutes; c__Clostridia; o__Clostridia; f__HungateiClostridiaceae; g__Saccharofermentans*
**BCG_138**	**757**	*p__Firmicutes; c__Clostridia; o__Christensenellales; f__Christensenellaceae ; g__Christensenellaceae _R-7_group; s__uncultured_Christensenella*<br>*p__Bacteroidota; c__Bacteroidia; o__Bacteroidales; f__Bacteroidales_UCG-001; g__Bacteroidales_UCG-001; s__uncultured_Bacteroidales*
**BCG_139**	**766**	*d__Bacteria*<br>*p__Firmicutes; c__Clostridia; o__Oscillospirales; f__UCG-010; g__UCG-010*

**BCG_141**	**708**	*p__Firmicutes; c__Negativicutes; o__Acidaminococcales; f__Acidaminococcaceae; g__Phascolarctobacterium; s__wallaby_gut*<br>*p__Firmicutes; c__Negativicutes; o__Acidaminococcales; f__Acidaminococcaceae; g__Phascolarctobacterium*
**BCG_142**	**726**	*p__Bacteroidota; c__Bacteroidia; o__Bacteroidales; f__Rikenellaceae; g__hoa5-07d05_gut_group; s__uncultured_bacterium*<br>*p__Bacteroidota; c__Bacteroidia; o__Bacteroidales; f__p-251-o5; g__p-251-o5; s__uncultured_Bacteroidales*
**ASV_31**	**708**	*p__Bacteroidota; c__Bacteroidia; o__Bacteroidales; f__Prevotellaceae; g__Prevotella*
**ASV_48**	**771**	*p__Bacteroidota; c__Bacteroidia; o__Bacteroidales; f__p-251-o5; g__p-251-o5; s__uncultured_bacterium*
**ASV_135**	**760**	*p__Bacteroidota; c__Bacteroidia; o__Bacteroidales; f__Rikenellaceae*
**ASV_382**	**742**	*p__Verrucomicrobiota; c__Kiritimatiellae; o__WCHB1-41; f__WCHB1-41; g__WCHB1-41; s__uncultured_prokaryote*

[1] Differential abundance was analyzed by Analysis of Composition of Microbes (ANCOM) in Qiime 2 (v.2020.8) [20,110].

[2] The ASV were grouped into BCG using Sparce Cooccurrence Network Investigation for Compositional Data [108] in Qiime 2 (v.2020.8).

[3] For ANCOM, $H_{0(ij)}$: mean(log[x_i/x_j) = mean(log[y_i/y_j). The W values indicate the number of times $H_{0(ij)}$ is rejected for the *ith* species.

[4] Taxonomic assignment was conducted using the most recent SILVA database (SSU 138).
