# Supplementary material for: Fecal microbiome of horses transitioning between warm-season and cool-season grass pasture within integrated rotational grazing systems
Source: Anim Microbiome. 2022 Jun 21;4:41. doi: 10.1186/s42523-022-00192-x (PMC9210719; doi:10.1186/s42523-022-00192-x)
Supplement: Supplementary file 3 — Additional file 3: Relative abundance of the twelve most abundant BCG that differed by horse. [file 42523_2022_192_MOESM3_ESM.pdf]

a.

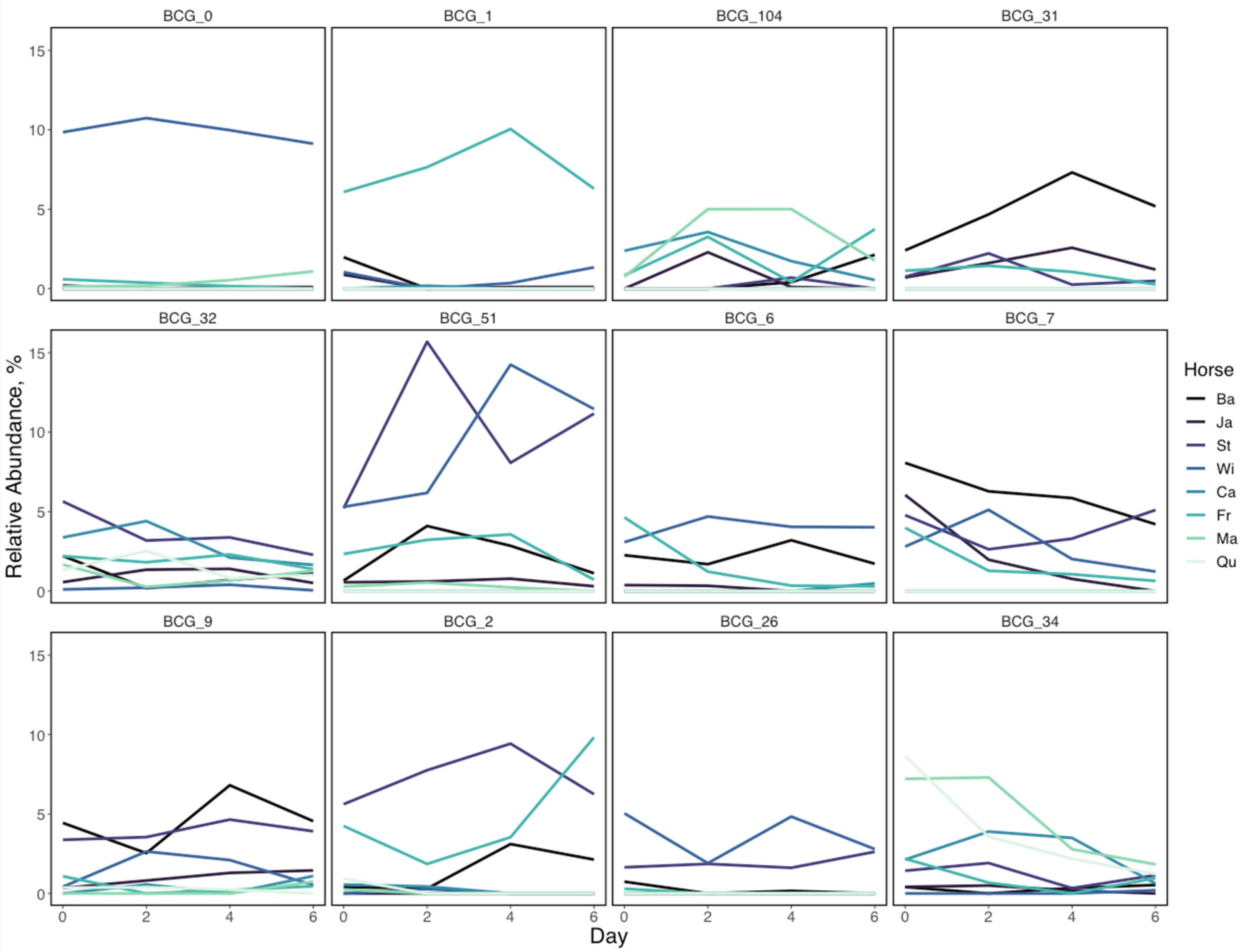
BCG_0
BCG_1
BCG_104
BCG_31
BCG_32
BCG_51
BCG_6
BCG_7
BCG_9
BCG_2
BCG_26
BCG_34
Relative Abundance, %
Day
Horse
Ba
Ja
St
Wi
Ca
Fr
Ma
Qu

b.

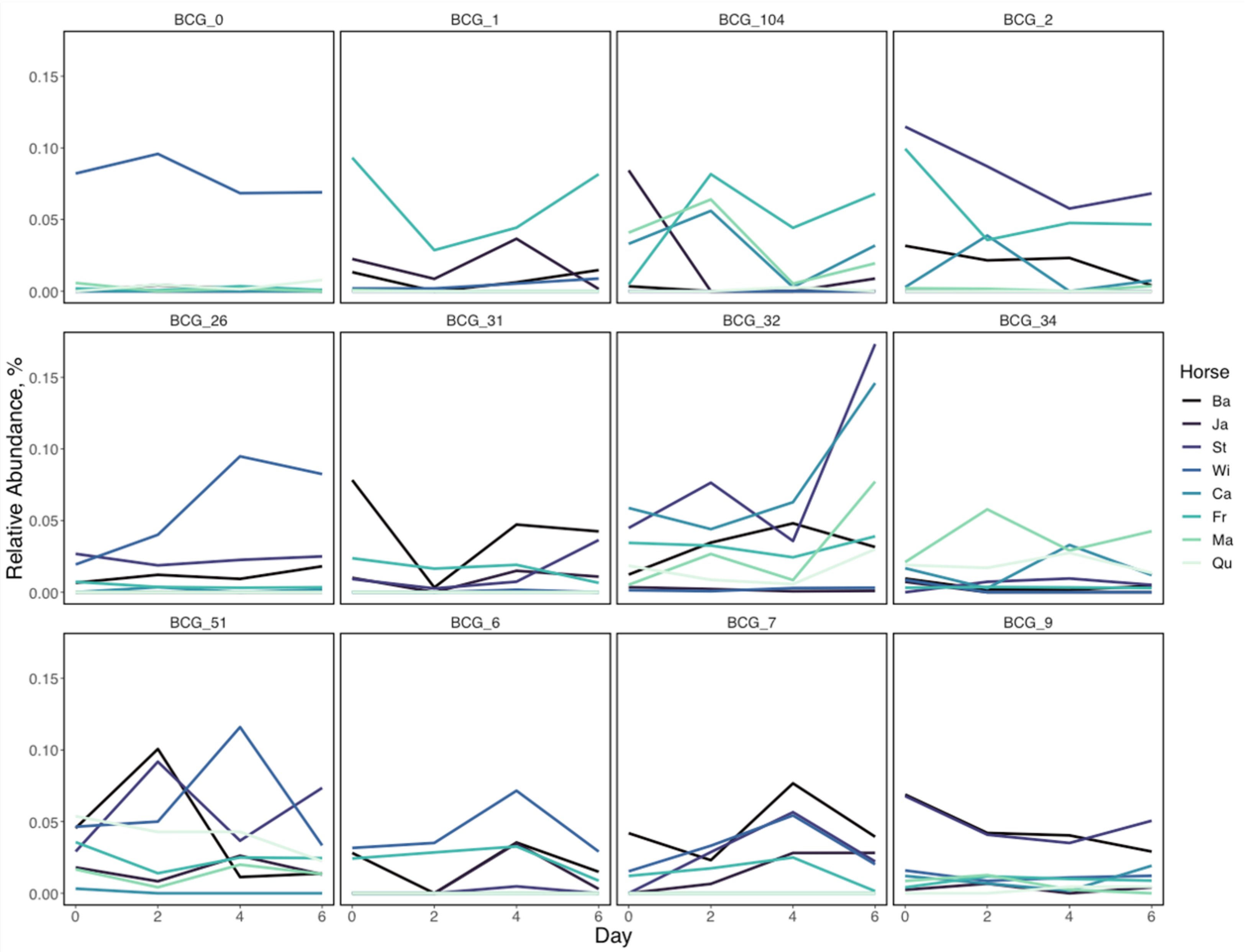

BCG_0
BCG_1
BCG_104
BCG_2
BCG_26
BCG_31
BCG_32
BCG_34
BCG_51
BCG_6
BCG_7
BCG_9
0.15
0.10
0.05
0.00
Relative Abundance, %
0
2
4
6
Day
Horse
Ba
Ja
St
Wi
Ca
Fr
Ma
Qu

Additional File 3. Fluctuations in relative abundances of bacteria differing by horse. Relative abundance most abundant bacterial co-abundance groups (BCG) identified as differentially abundant ($W \geq 704$) across horses are shown within transitions between (a) cool-season grass to warm-season grass pasture and (b) warm-season to cool-season grass based on Analysis of Composition of Microbes (ANCOM) in Qiime 2 (v.2020.8). Each BCG is represented by an individual facet grid. Individual horses are designated with two-letter abbreviations and arranged such that horses on the darker end of the color spectrum were those assigned to the bermudagrass integrated rotational grazing system (n=4) and horses on the lighter end of the color spectrum were those assigned to the crabgrass integrated system (n=4).
