# Supplementary material for: Fecal microbiome of horses transitioning between warm-season and cool-season grass pasture within integrated rotational grazing systems
Source: Anim Microbiome. 2022 Jun 21;4:41. doi: 10.1186/s42523-022-00192-x (PMC9210719; doi:10.1186/s42523-022-00192-x)
Supplement: Supplementary file 4 — Additional file 4: Genera groups and/or ungrouped genera identified as differentially abundant by grazing system, transition, day (within each grazing system for each transition) and horse. [file 42523_2022_192_MOESM4_ESM.pdf]

Additional File 4. Genera groups[1] and/or ungrouped genera identified as differentially abundant[2].

Variable	Genera Group [Genera]	Difference	W[3]
**Grazing System[4]**	Group 5 [Lachnospiraceae UCG-009; Prevotellaceae Ga6A1;, and an uncultured genus within Marinifilaceae]	More abundant in BRS	86
**Transition[5]**	Group 15 [Sphaerocheata; gir-aah93h0 (within the fir-aah93h0 family of Bacteroidales)]	More abundant in W-C	54
**Day (CRS; C-W)**	------	------	
**Day (CRS; W-C)**	Sarcinia	Increased over time	55
**Day (BRS; C-W)**	------	------	
**Day (CRS; W-C)**	Bacillus	Decreased over time	31
**Horse**	Group 1 [Methanocorpusculum; Bacteriodales UCG-001; Rikenellaceae RC9 gut group; an unclassified genus within Rikenellaceae; and UCG-010 (within the UCG-010 family of Oscillospirales)]	Varied across horses (n=8)	86
	Group 5 [Lachnospiraceae UCG-009; Prevotellaceae Ga6A1; and an uncultured genus within Marinifilaceae]	Varied across horses (n=8)	86
	Group 8 [MVP-15 (within MVP-15 family, order, and class of Spirochaetota); F082 (within the F082 family of Bacteroidales); and an unclassified genus within an unclassified family and order of Alphaproteobacteria)	Varied across horses (n=8)	75
	Group 17 [Schwartzia, Alistipes]	Varied across horses (n=8)	81
	Group 23 [COB P4-1 termite group and M2PB4-65 termite group]	Varied across horses (n=8)	70
	Group 30 [an unclassified genus within Ethanoligenenaceae; an unclassified genus within an unclassified family, order, class, and phylum of Bacteria]	Varied across horses (n=8)	86

Lachnospiraceae XPB1014 group	Varied across horses (n=8)	83
Candidatus Soleaferra (within Ruminococcaceae)	Varied across horses (n=8)	72

[1]Genera were grouped using Sparse Co-Occurrence Network Investigation for Compositional Data (SCNIC) in Qiime 2 (v.2020.8) (Boylen et al., 2019; Shaffer et al., 2020). Full genera grouping can be found in Additional File 3. After SCNIC, 56 genera remained ungrouped, 4 of which were differentially abundant either by day or by horse.

[2] Differential abundance was analyzed by Analysis of Composition of Microbes in Qiime 2 (v.2020.8) (Mandal et al., 2015; Boylen et al., 2019).

[3] For ANCOM, $H_{0(ij)}$: mean(log[x_i/x_j) = mean(log[y_i/y_j). Strength of the ANCOM statistical test is denoted by W values, which indicate the number of times $H_{0(ij)}$ is rejected for the *ith* species.

[4] Grazing systems: bermudagrass integrated rotational system (BRS); crabgrass integrated rotational system (CRS).

[5] Transitions: cool-season grass to warm-season grass (C-W); warm-season grass to cool-season grass (W-C).
