# Supplementary material for: Fecal microbiome of horses transitioning between warm-season and cool-season grass pasture within integrated rotational grazing systems
Source: Anim Microbiome. 2022 Jun 21;4:41. doi: 10.1186/s42523-022-00192-x (PMC9210719; doi:10.1186/s42523-022-00192-x)
Supplement: Supplementary file 5 — Additional file 5: Analysis comparison of grouping amplicon sequence variants into bacterial co-abundance groups (BCG) versus genus-level groupings. [file 42523_2022_192_MOESM5_ESM.pdf]

Additional File 5. Analysis comparison of grouping amplicon sequence variants into bacterial co-abundance groups (BCG) versus genus-level groupings[1].

Test	Variable	Grouping	Value
**Random Forest Classification, accuracy[2]**	Horse	BCG	0.92 ± 0.05
		Genera Group	0.80 ± 0.09
	Grazing System	BCG	0.95 ± 0.06
		Genera Group	0.84 ± 0.11
	Transition	BCG	0.97 ± 0.06
		Genera Group	0.84 ± 0.07
	Day (C-W)	BCG	0.25 ± 0.13
		Genera Group	0.13 ± 0.12
	Day (W-C)	BCG	0.22 ± 0.18
		Genera Group	0.25 ± 0.13
**ANCOM, %[3]**	Horse	BCG	32
		Genera Group	22
	Grazing System	BCG	7
		Genera Group	2
	Transition	BCG	1
		Genera Group	0.1
	Day (both transitions)	BCG	2
		Genera Group	1

[1]Amplicon sequence variants and genera were grouped using Sparse Co-Occurrence Network Investigation for Compositional Data (SCNIC) in Qiime 2 (v.2020.8) (Boylen et al., 2019; Shaffer et al., 2020).

[2] Random forest classifiers with nested cross validation (q2-sample-classifier plugin) were applied to determine if metadata variables (grazing system, field, day, horse) could be predicted based on microbial composition – using abundance profiles of either BCG or genera groups (Pedregosa et al., 2011; Bukolich et al., 2018a). Data presented represent accuracies of random forest classifiers (scale: 0.0 – 1.0).

[3] Differential abundance was analyzed by Analysis of Composition of Microbes (ANCOM) (Mandal et al., 2015; Boylen et al., 2019). Data presented indicate the percentage of the total microbial community represented by features (either BCG or genera groups) identified as differential abundant.
