# Supplementary material for: Fecal microbiome of horses transitioning between warm-season and cool-season grass pasture within integrated rotational grazing systems
Source: Anim Microbiome. 2022 Jun 21;4:41. doi: 10.1186/s42523-022-00192-x (PMC9210719; doi:10.1186/s42523-022-00192-x)
Supplement: Supplementary file 6 — Additional file 6: Monthly average temperatures and precipitation totals across the study period and historical averages. [file 42523_2022_192_MOESM6_ESM.pdf]

Additional File 6. Weather data[1].

Transition/Grazing System[2]	Max Temperature, °C	Total Precipitation, cm
**C-W**		
BRS	23.52	8.05
CRS	31.76	1.32
**W-C**		
BRS	25.46	3.20
CRS	31.11	0.71

[1] Weather data were obtained for the New Brunswick Station through the Office of the New Jersey State Climatologist website [80]. Data are presented as averages over the first six days of the transitions.

[2] C-W: transitions from cool-season to warm-season grass; W-C: warm-season to cool-season grass; BRS: bermudagrass integrated rotational grazing system; CRS: crabgrass integrated system.
