# Supplementary material for: Fecal microbiome of horses transitioning between warm-season and cool-season grass pasture within integrated rotational grazing systems
Source: Anim Microbiome. 2022 Jun 21;4:41. doi: 10.1186/s42523-022-00192-x (PMC9210719; doi:10.1186/s42523-022-00192-x)
Supplement: Supplementary file 7 — Additional file 7: Diagram of experimental design and sampling protocol. [file 42523_2022_192_MOESM7_ESM.pdf]

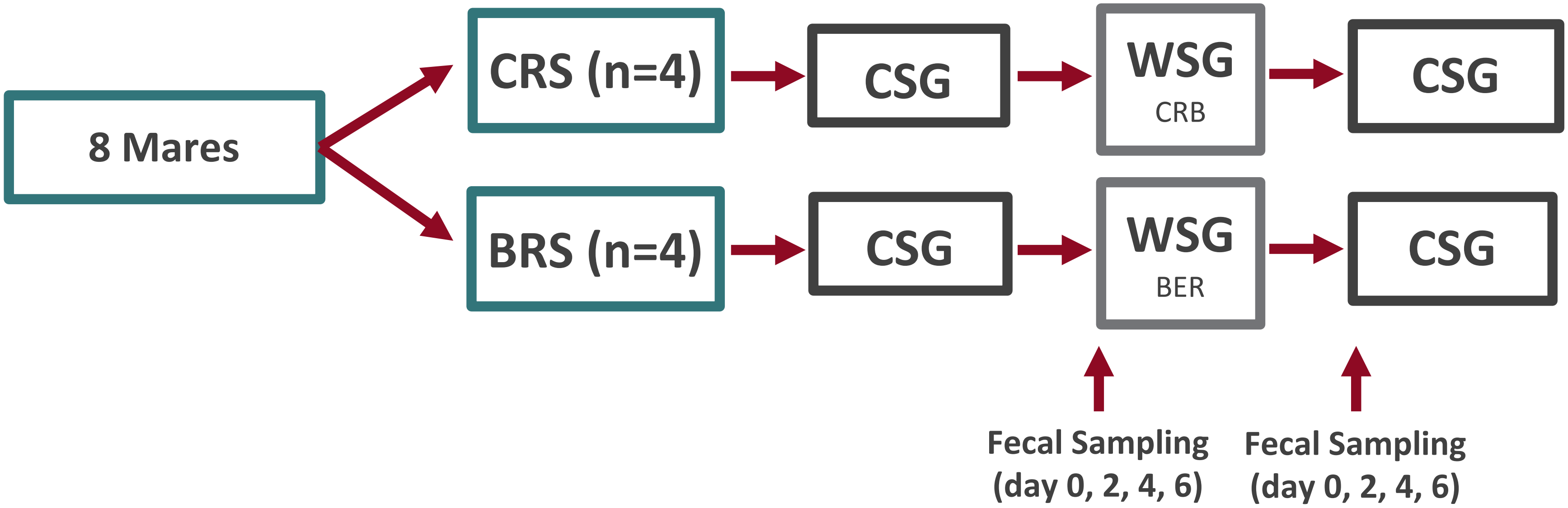

Additional File 7. Diagram of experimental design and sampling protocol. Eight healthy standardbred mares were randomly assigned to one of two grazing systems (n=4 per system), with one system containing crabgrass (CRB) and the other containing bermudagrass (BER) as the warm-season grass (WSG). Horses grazed on cool-season grass (CSG) for a minimum of 21 days prior to transitioning to WSG and then grazes WSG for 17-21 days (based on forage availability) before transitioning back to CSG. Manual grab fecal samples were collected on days 0, 2, 4, 6 of each transition.
