# Supplementary material for: Fecal microbiome of horses transitioning between warm-season and cool-season grass pasture within integrated rotational grazing systems
Source: Anim Microbiome. 2022 Jun 21;4:41. doi: 10.1186/s42523-022-00192-x (PMC9210719; doi:10.1186/s42523-022-00192-x)
Supplement: Supplementary file 8 — Additional file 8: Code used for sequence and statistical analysis in Qiime 2 (v.2020.8) and R (v. 4.0.2). [file 42523_2022_192_MOESM8_ESM.pdf]

Additional File 7. Code used for sequence and statistical analysis in Qiime 2 (v.2020.8) and R (v. 4.0.2).

QIIME 2 Code:

```
qiime tools import \
  --type 'SampleData[SequencesWithQuality]' \
  --input-path adapted/fastq-manifest-rot.tsv \
  --output-path adapted/rot_demux.qza \
  --input-format PairedEndFastqManifestPhred33

qiime demux summarize \
  --i-data adapted/rot_demux.qza \
  --o-visualization adapted/rot_demux.qzv

qiime dada2 denoise-single \
  --i-demultiplexed-seqs rot_demux.qza \
  --p-trim-left-f 20 \
  --p-trim-left-r 20 \
  --p-trunc-len-f 260 \
  --p-trunc-len-f 200 \
  --o-representative-sequences rep-seqs-rot.qza \
  --o-table table-rot.qza \
  --o-denoising-stats stats-rot2.qza

qiime metadata tabulate \
  --m-input-file stats-rot2.qza \
  --o-visualization stats-rot2.qzv

qiime feature-table tabulate-seqs \
  --i-data rep-seqs-rot.qza \
  --o-visualization rep-seqs-rot.qzv

qiime phylogeny align-to-tree-mafft-fasttree \
  --i-sequences rep-seqs-rot.qza \
  --o-alignment aligned-rep-seqs-rot.qza \
  --o-masked-alignment masked-aligned-rep-seqs-rot.qza \
  --o-tree unrooted-tree_rot.qza\
  --o-rooted-tree rooted-tree_rot.qza
qiime feature-table filter-features \
  --i-table table-rot.qza \
  --p-min-frequency 9 \
  --o-filtered-table feature-frequency-filtered-table-rot.qza

qiime feature-table filter-features \
  --i-table feature-frequency-filtered-table-rot.qza \
  --p-min-samples 4 \
  --o-filtered-table filtered-table-rot.qza
```

```
qiime feature-table summarize \
  --i-table filtered-table-rot.qza \
  --o-visualization table-rot.qzv \
  --m-sample-metadata-file 2018_Rot.tsv

qiime diversity core-metrics-phylogenetic \
  --i-phylogeny rooted-tree_rot.qza \
  --i-table filtered-table-rot.qza \
  --p-sampling-depth 3700 \
  --m-metadata-file 2018_Rot.tsv \
  --output-dir core-metrics-results

qiime diversity alpha-rarefaction \
  --i-table filtered-table-rot.qza \
  --i-phylogeny rooted-tree_rot.qza \
  --p-max-depth 4000 \
  --m-metadata-file 2018_Rot.tsv \
  --o-visualization alpha-rarefaction_rot.qzv
```

****See R code for statistical analysis of alpha diversity metrics.

```
qiime diversity beta-group-significance \
  --i-distance-matrix core-metrics-results/unweighted_unifrac_distance_matrix.qza \
  --m-metadata-file 2018_Rot.tsv \
  --m-metadata-column system\
  --o-visualization core-metrics-results/unweighted-unifrac-system-significance.qzv \
  --p-method permdisp

qiime diversity beta-group-significance \
  --i-distance-matrix core-metrics-results/unweighted_unifrac_distance_matrix.qza \
  --m-metadata-file 2018_Rot.tsv \
  --m-metadata-column transition\
  --o-visualization core-metrics-results/unweighted-unifrac-transition-significance.qzv \
  --p-method permdisp
qiime diversity beta-group-significance \
  --i-distance-matrix core-metrics-results/unweighted_unifrac_distance_matrix.qza \
  --m-metadata-file 2018_Rot.tsv \
  --m-metadata-column day\
  --o-visualization core-metrics-results/unweighted-unifrac-day-significance.qzv \
  --p-method permdisp

qiime diversity beta-group-significance \
  --i-distance-matrix core-metrics-results/unweighted_unifrac_distance_matrix.qza \
  --m-metadata-file 2018_Rot.tsv \
  --m-metadata-column horse\
  --o-visualization core-metrics-results/unweighted-unifrac-horse-significance.qzv \
  --p-method permdisp

qiime diversity beta-group-significance \
  --i-distance-matrix core-metrics-results/weighted_unifrac_distance_matrix.qza \
  --m-metadata-file 2018_Rot.tsv \
```

```
 --m-metadata-column system\
 --o-visualization core-metrics-results/weighted-unifrac-system-significance.qzv \
 --p-method permdisp

qiime diversity beta-group-significance \
 --i-distance-matrix core-metrics-results/weighted_unifrac_distance_matrix.qza \
 --m-metadata-file 2018_Rot.tsv \
 --m-metadata-column transition\
 --o-visualization core-metrics-results/weighted-unifrac-transition-significance.qzv \
 --p-method permdisp
qiime diversity beta-group-significance \
 --i-distance-matrix core-metrics-results/weighted_unifrac_distance_matrix.qza \
 --m-metadata-file 2018_Rot.tsv \
 --m-metadata-column day\
 --o-visualization core-metrics-results/weighted-unifrac-day-significance.qzv \
 --p-method permdisp

qiime diversity beta-group-significance \
 --i-distance-matrix core-metrics-results/weighted_unifrac_distance_matrix.qza \
 --m-metadata-file 2018_Rot.tsv \
 --m-metadata-column horse\
 --o-visualization core-metrics-results/weighted-unifrac-horse-significance.qzv \
 --p-method permdisp

qiime diversity beta-group-significance \
 --i-distance-matrix core-metrics-results/bray_curtis_distance_matrix.qza \
 --m-metadata-file 2018_Rot.tsv \
 --m-metadata-column system\
 --o-visualization core-metrics-results/ bray_curtis-system-significance.qzv \
 --p-method permdisp

qiime diversity beta-group-significance \
 --i-distance-matrix core-metrics-results/ bray_curtis_distance_matrix.qza \
 --m-metadata-file 2018_Rot.tsv \
 --m-metadata-column transition\
 --o-visualization core-metrics-results/bray_curtis-transition-significance.qzv \
 --p-method permdisp

qiime diversity beta-group-significance \
 --i-distance-matrix core-metrics-results/ bray_curtis_distance_matrix.qza \
 --m-metadata-file 2018_Rot.tsv \
 --m-metadata-column day\
 --o-visualization core-metrics-results/ bray_curtis-day-significance.qzv \
 --p-method permdisp

qiime diversity beta-group-significance \
 --i-distance-matrix core-metrics-results/ bray_curtis_distance_matrix.qza \
 --m-metadata-file 2018_Rot.tsv \
 --m-metadata-column horse\
 --o-visualization core-metrics-results/ bray_curtis-horse-significance.qzv \
 --p-method permdisp
```

```
qiime diversity beta-group-significance \
  --i-distance-matrix core-metrics-results/jaccard_distance_matrix.qza \
  --m-metadata-file 2018_Rot.tsv \
  --m-metadata-column system\
  --o-visualization core-metrics-results/ jaccard-system-significance.qzv \
  --p-method permdisp

qiime diversity beta-group-significance \
  --i-distance-matrix core-metrics-results/ jaccard_distance_matrix.qza \
  --m-metadata-file 2018_Rot.tsv \
  --m-metadata-column transition\
  --o-visualization core-metrics-results/unweighted-unifrac-transition-significance.qzv \
  --p-method permdisp
qiime diversity beta-group-significance \
  --i-distance-matrix core-metrics-results/ jaccard_distance_matrix.qza \
  --m-metadata-file 2018_Rot.tsv \
  --m-metadata-column day\
  --o-visualization core-metrics-results/ jaccard-day-significance.qzv \
  --p-method permdisp

qiime diversity beta-group-significance \
  --i-distance-matrix core-metrics-results/ jaccard_distance_matrix.qza \
  --m-metadata-file 2018_Rot.tsv \
  --m-metadata-column horse\
  --o-visualization core-metrics-results/ jaccard-horse-significance.qzv \
  --p-method permdisp

qiime diversity adonis \
  --i-distance-matrix unweighted_unifrac_distance_matrix.qza \
  --m-metadata-file 2018_Rot.tsv \
  --o-visualization unweighted_adonis.qzv \
  --p-formula "system*transition*day*horse"

qiime diversity adonis \
  --i-distance-matrix weighted_unifrac_distance_matrix.qza \
  --m-metadata-file 2018_Rot.tsv \
  --o-visualization weighted_adonis.qzv \
  --p-formula "system*transition*day*horse "

qiime diversity adonis \
  --i-distance-matrix jaccard_distance_matrix.qza \
  --m-metadata-file 2018_Rot.tsv \
  --o-visualization jaccard_adonis.qzv \
  --p-formula "system*transition*day*horse "

qiime diversity adonis \
  --i-distance-matrix bray_curtis_distance_matrix.qza \
  --m-metadata-file 2018_Rot.tsv \
  --o-visualization bray_adonis.qzv \
  --p-formula "system*transition*day*horse "
```

```
qiime feature-classifier classify-sklearn \
  --i-classifier silva-classifier.qza \
  --i-reads rep-seqs-rot.qza \
  --o-classification rot-taxonomy.qza

qiime metadata tabulate \
  --m-input-file rot-taxonomy.qza \
  --o-visualization rot-taxonomy.qzv

qiime feature-table filter-samples \
  --i-table filtered-table-rot.qza \
  --m-metadata-file 2018_Rot.tsv \
  --p-where "[Rot]='CW'" \
  --o-filtered-table cw-table-rot.qza

qiime feature-table filter-samples \
  --i-table filtered-table-rot.qza \
  --m-metadata-file 2018_Rot.tsv \
  --p-where "[Rot]='WC'" \
  --o-filtered-table wc-table-rot.qza

qiime taxa collapse \
  --i-table cw-table-rot.qza \
  --i-taxonomy rot-taxonomy.qza \
  --p-level 2 \
  --o-collapsed-table cw-rot-table-l2.qza

qiime feature-table summarize \
  --i-table cw-rot-table-l2.qza \
  --o-visualization cw-rot-table-l2.qzv \
  --m-sample-metadata-file 2018_Rot.tsv

qiime feature-table relative-frequency \
  --i-table cw-rot-table-l2.qza \
  --o-relative-frequency-table cw-rot-table-l2-rel.qza

qiime tools export --input-path cw-rot-table-l2-rel.qza --output-path exported_tables_l2_rot_cw
biom convert -i exported_tables_l2_rot_ cw/feature-table.biom -o exported_tables_l2_rot_ cw/feature-
table.tsv --to-tsv
biom head -i exported_tables_l2_rot_ cw/feature-table.tsv

qiime tools export --input-path wc-rot-table-l2-rel.qza --output-path exported_tables_l2_rot_wc
biom convert -i exported_tables_l2_rot_ wc/feature-table.biom -o exported_tables_l2_rot_ wc/feature-
table.tsv --to-tsv
biom head -i exported_tables_l2_rot_ wc/feature-table.tsv
```

****See R code for statistical analysis of phylum-level abundance.

```
qiime SCNIC sparcc-filter \
  --i-table filtered-table-rot.qza \
```

```
  --o-table-filtered scnic_table_rot.qza

qiime SCNIC calculate-correlations \
  --i-table scnic_table_rot.qza \
  --p-method spearman \
  --o-correlation-table scnic-correls.qza \
  --verbose

qiime SCNIC build-correlation-network-r \
  --i-correlation-table scnic-correls.qza \
  --p-min-val .4 \
  --o-correlation-network scnic_net.qza

qiime SCNIC build-correlation-network-p \
  --i-correlation-table scnic-correls.qza \
  --p-max-val .05 \
  --o-correlation-network scnic_net_sig.qza

qiime SCNIC make-modules-on-correlations \
  --i-correlation-table scnic-correls.qza \
  --i-feature-table filtered-table-rot.qza \
  --p-min-r .4 \
  --o-collapsed-table scnic.collapsed.qza \
  --o-correlation-network scnic.modules.qza \
  --o-module-membership scnic_membership.qza

qiime metadata tabulate \
  --m-input-file scnic_membership.qza \
  --o-visualization scnic_membership.qzv

qiime feature-table relative-frequency \
  --i-table manuscript/scnic.collapsed.qza \
  --o-relative-frequency-table manuscript/scnic_table_rel.qza

qiime sample-classifier classify-samples-ncv \
  --i-table manuscript/ scnic.collapsed.qza \
  --m-metadata-file 2018_Rot.tsv \
  --m-metadata-column system \
  --p-estimator RandomForestClassifier \
  --p-n-estimators 200 \
  --p-random-state 123 \
  --o-predictions system-predictions-ncv.qza \
  --o-probabilities system-probabilities-ncv.qza \
  --o-feature-importance system-importance-ncv.qza \
  --verbose

qiime sample-classifier classify-samples-ncv \
  --i-table scnic.collapsed.qza \
  --m-metadata-file 2018_Rot.tsv \
  --m-metadata-column transition \
  --p-estimator RandomForestClassifier \
```

```
  --p-n-estimators 200 \
  --p-random-state 123 \
  --o-predictions transition-predictions-ncv.qza \
  --o-probabilities transition-probabilities-ncv.qza \
  --o-feature-importance transition-importance-ncv.qza \
  --verbose

qiime sample-classifier classify-samples-ncv \
  --i-table scnic.collapsed.qza \
  --m-metadata-file 2018_Rot.tsv \
  --m-metadata-column horse \
  --p-estimator RandomForestClassifier \
  --p-n-estimators 200 \
  --p-random-state 123 \
  --o-predictions horse-predictions-ncv.qza \
  --o-probabilities horse-probabilities-ncv.qza \
  --o-feature-importance horse-importance-ncv.qza \
  --verbose

qiime feature-table filter-samples \
  --i-table scnic.collapsed.qza \
  --m-metadata-file 2018_rot.tsv \
  --p-where "[Rot]='CW'" \
  --o-filtered-table manuscript/cw-scnic-table.qza

qiime feature-table filter-samples \
  --i-table scnic.collapsed.qza \
  --m-metadata-file 2018_rot.tsv \
  --p-where "[Rot]='WC'" \
  --o-filtered-table wc-scnic-table.qza

qiime feature-table filter-samples \
  --i-table wc-scnic-table.qza \
  --m-metadata-file 2018_Rot.tsv \
  --p-where "[Field]='BRS'" \
  --o-filtered-table wc_brs_scnic_table.qza

qiime feature-table filter-samples \
  --i-table wc-scnic-table.qza \
  --m-metadata-file 2018_Rot.tsv \
  --p-where "[Field]='CRS'" \
  --o-filtered-table wc_crs_scnic_table.qza

qiime feature-table filter-samples \
  --i-table cw-scnic-table.qza \
  --m-metadata-file 2018_Rot.tsv \
  --p-where "[Field]='BRS'" \
  --o-filtered-table cw_brs_scnic_table.qza

qiime feature-table filter-samples \
  --i-table cw-scnic-table.qza \
```

```
  --m-metadata-file 2018_Rot.tsv \
  --p-where "[Field]='CRS'" \
  --o-filtered-table cw_crs_scnic_table.qza

qiime sample-classifier classify-samples-ncv \
  --i-table wc-scnic-table.qza \
  --m-metadata-file 2018_Rot.tsv \
  --m-metadata-column day \
  --p-estimator RandomForestClassifier \
  --p-n-estimators 200 \
  --p-random-state 123 \
  --o-predictions scnic-wc_day_predictions-ncv.qza \
  --o-probabilities scnic-wc_day_probabilities-ncv.qza \
  --o-feature-importance scnic-wc_day_importance-ncv.qza \
  --verbose

qiime sample-classifier classify-samples-ncv \
  --i-table CW-scnic-table.qza \
  --m-metadata-file 2018_Rot.tsv \
  --m-metadata-column day \
  --p-estimator RandomForestClassifier \
  --p-n-estimators 200 \
  --p-random-state 123 \
  --o-predictions scnic-CW_day_predictions-ncv.qza \
  --o-probabilities scnic-CW_day_probabilities-ncv.qza \
  --o-feature-importance scnic-CWC_day_importance-ncv.qza \
  --verbose

qiime composition add-pseudocount \
  --i-table scnic.collapsed.qza \
  --o-composition-table comp_scnic.collapsed.qza

qiime composition add-pseudocount \
  --i-table cw_crs_scnic_table.qza \
  --o-composition-table comp_cw_crs_scnic_table.qza

qiime composition add-pseudocount \
  --i-table cw_brs_scnic_table.qza \
  --o-composition-table comp_cw_brs_scnic_table.qza

qiime composition add-pseudocount \
  --i-table wc_crs_scnic_table.qza \
  --o-composition-table comp_wc_crs_scnic_table.qza

qiime composition add-pseudocount \
  --i-table wc_brs_scnic_table.qza \
  --o-composition-table comp_wc_brs_scnic_table.qza

qiime composition ancom \
  --i-table comp_scnic.collapsed.qza \
  --m-metadata-file 2018_Rot.tsv \
```

```
  --m-metadata-column system \
  --o-visualization scnic_ancom_system.qzv

qiime composition ancom \
  --i-table comp_scnic.collapsed.qza \
  --m-metadata-file 2018_Rot.tsv \
  --m-metadata-column transition \
  --o-visualization scnic_ancom_transition.qzv

qiime composition ancom \
  --i-table comp_scnic.collapsed.qza \
  --m-metadata-file 2018_Rot.tsv \
  --m-metadata-column horse \
  --o-visualization scnic_ancom_horse.qzv

qiime composition ancom \
  --i-table comp_cw_crs_scnic_table.qza \
  --m-metadata-file 2018_Rot.tsv \
  --m-metadata-column day \
  --o-visualization scnic_ancom_cw_crs_day.qzv

qiime composition ancom \
  --i-table comp_cw_brs_scnic_table.qza \
  --m-metadata-file 2018_Rot.tsv \
  --m-metadata-column day \
  --o-visualization scnic_ancom_cw_brs_day.qzv

qiime composition ancom \
  --i-table comp_wc_crs_scnic_table.qza \
  --m-metadata-file 2018_Rot.tsv \
  --m-metadata-column day \
  --o-visualization scnic_ancom_wc_crs_day.qzv

qiime composition ancom \
  --i-table comp_wc_brs_scnic_table.qza \
  --m-metadata-file 2018_Rot.tsv \
  --m-metadata-column day \
  --o-visualization scnic_ancom_wc_brs_day.qzv

qiime taxa collapse \
  --i-table filter-table-rot.qza \
  --i-taxonomy filter-taxonomy.qza \
  --p-level 6 \
  --o-collapsed-table genus_table_rot.qza

qiime feature-table summarize \
  --i-table genus_table_rot.qza \
  --o-visualization genus_table_rot.qzv \
  --m-sample-metadata-file 2018_Rot.tsv

qiime feature-table relative-frequency \
```

```
  --i-table genus_table_rot.qza \
  --o-relative-frequency-table genus_table_rot-rel.qza

qiime tools export --input-path genus_table_rot-rel.qza --output-path exported_tables_genus
biom convert -i exported_tables_genus/feature-table.biom -o exported_tables_genus/feature-table.tsv --to-tsv
biom head -i exported_tables_genus/feature-table.tsv

qiime SCNIC sparcc-filter \
  --i-table genus_table_rot.qza \
  --o-table-filtered scnic_genus_rot.qza

qiime SCNIC calculate-correlations \
  --i-table scnic_genus_rot.qza \
  --p-method spearman \
  --o-correlation-table scnic-correls-genus.qza \
  --verbose

qiime SCNIC build-correlation-network-r \
  --i-correlation-table scnic-correls-genus.qza \
  --p-min-val .4 \
  --o-correlation-network scnic_net-genus.qza

qiime SCNIC build-correlation-network-p \
  --i-correlation-table scnic-correls-genus.qza \
  --p-max-val .05 \
  --o-correlation-network scnic_net_sig-genus.qza

qiime SCNIC make-modules-on-correlations \
  --i-correlation-table scnic-correls-genus.qza \
  --i-feature-table genus_table_rot.qza \
  --p-min-r .4 \
  --o-collapsed-table scnic.collapsed-genus.qza \
  --o-correlation-network scnic.modules-genus.qza \
  --o-module-membership scnic_membership-genus.qza

qiime metadata tabulate \
  --m-input-file scnic_membership-genus.qza \
  --o-visualization scnic_membership-genus.qzv

qiime feature-table relative-frequency \
  --i-table manuscript/scnic.collapsed-genus.qza \
  --o-relative-frequency-table manuscript/scnic_genus_rel.qza

qiime sample-classifier classify-samples-ncv \
  --i-table manuscript/ scnic.collapsed-genus.qza \
  --m-metadata-file 2018_Rot.tsv \
  --m-metadata-column system \
  --p-estimator RandomForestClassifier \
  --p-n-estimators 200 \
  --p-random-state 123 \
```

```
  --o-predictions system-predictions-ncv-genus.qza \
  --o-probabilities system-probabilities-ncv-genus.qza \
  --o-feature-importance system-importance-ncv-genus.qza \
  --verbose

qiime sample-classifier classify-samples-ncv \
  --i-table scnic.collapsed-genus.qza \
  --m-metadata-file 2018_Rot.tsv \
  --m-metadata-column transition \
  --p-estimator RandomForestClassifier \
  --p-n-estimators 200 \
  --p-random-state 123 \
  --o-predictions transition-predictions-ncv-genus.qza \
  --o-probabilities transition-probabilities-ncv-genus.qza \
  --o-feature-importance transition-importance-ncv-genus.qza \
  --verbose

qiime sample-classifier classify-samples-ncv \
  --i-table scnic.collapsed-genus.qza \
  --m-metadata-file 2018_Rot.tsv \
  --m-metadata-column horse \
  --p-estimator RandomForestClassifier \
  --p-n-estimators 200 \
  --p-random-state 123 \
  --o-predictions horse-predictions-ncv-genus.qza \
  --o-probabilities horse-probabilities-ncv-genus.qza \
  --o-feature-importance horse-importance-ncv-genus.qza \
  --verbose

qiime feature-table filter-samples \
  --i-table scnic.collapsed-genus.qza \
  --m-metadata-file 2018_rot.tsv \
  --p-where "[Rot]='CW'" \
  --o-filtered-table manuscript/cw-scnic-table-genus.qza

qiime feature-table filter-samples \
  --i-table scnic.collapsed-genus.qza \
  --m-metadata-file 2018_rot.tsv \
  --p-where "[Rot]='WC'" \
  --o-filtered-table wc-scnic-table-genus.qza

qiime feature-table filter-samples \
  --i-table wc-scnic-table-genus.qza \
  --m-metadata-file 2018_Rot.tsv \
  --p-where "[Field]='BRS'" \
  --o-filtered-table wc_brs_scnic_table-genus.qza

qiime feature-table filter-samples \
  --i-table wc-scnic-table-genus.qza \
  --m-metadata-file 2018_Rot.tsv \
  --p-where "[Field]='CRS'" \
```

```
  --o-filtered-table wc_crs_scnic_table-genus.qza

qiime feature-table filter-samples \
  --i-table cw-scnic-table-genus.qza \
  --m-metadata-file 2018_Rot.tsv \
  --p-where "[Field]='BRS'" \
  --o-filtered-table cw_brs_scnic_table-genus.qza

qiime feature-table filter-samples \
  --i-table cw-scnic-table-genus.qza \
  --m-metadata-file 2018_Rot.tsv \
  --p-where "[Field]='CRS'" \
  --o-filtered-table cw_crs_scnic_table-genus.qza

qiime sample-classifier classify-samples-ncv \
  --i-table wc-scnic-table-genus.qza \
  --m-metadata-file 2018_Rot.tsv \
  --m-metadata-column day \
  --p-estimator RandomForestClassifier \
  --p-n-estimators 200 \
  --p-random-state 123 \
  --o-predictions scnic-wc_day_predictions-ncv-genus.qza \
  --o-probabilities scnic-wc_day_probabilities-ncv-genus.qza \
  --o-feature-importance scnic-wc_day_importance-ncv-genus.qza \
  --verbose

qiime sample-classifier classify-samples-ncv \
  --i-table CW-scnic-table-genus.qza \
  --m-metadata-file 2018_Rot.tsv \
  --m-metadata-column day \
  --p-estimator RandomForestClassifier \
  --p-n-estimators 200 \
  --p-random-state 123 \
  --o-predictions scnic-CW_day_predictions-ncv-genus.qza \
  --o-probabilities scnic-CW_day_probabilities-ncv-genus.qza \
  --o-feature-importance scnic-CWC_day_importance-ncv-genus.qza \
  --verbose

qiime composition add-pseudocount \
  --i-table scnic.collapsed-genus.qza \
  --o-composition-table comp_scnic.collapsed-genus.qza

qiime composition add-pseudocount \
  --i-table cw_crs_scnic_table-genus.qza \
  --o-composition-table comp_cw_crs_scnic_table-genus.qza

qiime composition add-pseudocount \
  --i-table cw_brs_scnic_table-genus.qza \
  --o-composition-table comp_cw_brs_scnic_table-genus.qza

qiime composition add-pseudocount \
```

```
  --i-table wc_crs_scnic_table-genus.qza \
  --o-composition-table comp_wc_crs_scnic_table-genus.qza

qiime composition add-pseudocount \
  --i-table wc_brs_scnic_table-genus.qza \
  --o-composition-table comp_wc_brs_scnic_table-genus.qza

qiime composition ancom \
  --i-table comp_scnic.collapsed-genus.qza \
  --m-metadata-file 2018_Rot.tsv \
  --m-metadata-column system \
  --o-visualization scnic_ancom_system-genus.qzv

qiime composition ancom \
  --i-table comp_scnic.collapsed-genus.qza \
  --m-metadata-file 2018_Rot.tsv \
  --m-metadata-column transition \
  --o-visualization scnic_ancom_transition-genus.qzv

qiime composition ancom \
  --i-table comp_scnic.collapsed-genus.qza \
  --m-metadata-file 2018_Rot.tsv \
  --m-metadata-column horse \
  --o-visualization scnic_ancom_horse-genus.qzv

qiime composition ancom \
  --i-table comp_cw_crs_scnic_table-genus.qza \
  --m-metadata-file 2018_Rot.tsv \
  --m-metadata-column day \
  --o-visualization scnic_ancom_cw_crs_day-genus.qzv

qiime composition ancom \
  --i-table comp_cw_brs_scnic_table-genus.qza \
  --m-metadata-file 2018_Rot.tsv \
  --m-metadata-column day \
  --o-visualization scnic_ancom_cw_brs_day-genus.qzv

qiime composition ancom \
  --i-table comp_wc_crs_scnic_table-genus.qza \
  --m-metadata-file 2018_Rot.tsv \
  --m-metadata-column day \
  --o-visualization scnic_ancom_wc_crs_day-genus.qzv

qiime composition ancom \
  --i-table comp_wc_brs_scnic_table-genus.qza \
  --m-metadata-file 2018_Rot.tsv \
  --m-metadata-column day \
  --o-visualization scnic_ancom_wc_brs_day-genus.qzv
```

R Code:

```
> observed_asvs$day<-as.factor(observed_otus$day)
> otus_lme<- lme(observed_otus~system*transition*day, random = list(Horse=~1, Rot=~Day),data=observed_otus)
> summary(asvs_lme)
> plot(asvs_lme)
> shapiro.test(resid(asvs_lme))
> anova(asvs_lme)
> lsmeans(asvs_lme,pairwise~ system*transition*day,adjust="tukey")
> lsmeans(asvs_lme,pairwise~ system*transition,adjust="tukey")
> lsmeans(asvs_lme,pairwise~ transition*day,adjust="tukey")
> lsmeans(asvs_lme,pairwise~ system,adjust="tukey")
> lsmeans(asvs_lme,pairwise~ transition,adjust="tukey")
> lsmeans(asvs_lme,pairwise~ day,adjust="tukey")
```

****Above code repeated for each diversity metric.

```
kruskal.test(Firmicutes~ Day, data = cw_phy)
kruskal.test(Firmicutes~ Day, data = wc_phy)
```

****Above code repeated for each phylum.
